# Mapping Molecular Recognition of β1,3-1,4-Glucans by a Surface Glycan-Binding Protein from the Human Gut Symbiont Bacteroides ovatus

**DOI:** 10.1128/Spectrum.01826-21

**Published:** 2021-11-24

**Authors:** Viviana G. Correia, Filipa Trovão, Benedita A. Pinheiro, Joana L. A. Brás, Lisete M. Silva, Cláudia Nunes, Manuel A. Coimbra, Yan Liu, Ten Feizi, Carlos M. G. A. Fontes, Barbara Mulloy, Wengang Chai, Ana Luísa Carvalho, Angelina S. Palma

**Affiliations:** a UCIBIO, Applied Molecular Biosciences Unit, Department of Chemistry, School of Science and Technology, NOVA University Lisbon, Caparica, Portugal; b Associate Laboratory i4HB—Institute for Health and Bioeconomy, School of Science and Technology, NOVA University Lisbon, Caparica, Portugal; c NZYTech Genes & Enzymes, Lisbon, Portugal; d Glycosciences Laboratory, Department of Metabolism, Digestion and Reproduction, Imperial College London, London, United Kingdom; e CICECO, Department of Chemistry, University of Aveiro, Aveiro, Portugal; f LAQV-REQUIMTE, Department of Chemistry, University of Aveiro, Aveiro, Portugal; g CIISA, Faculty of Veterinary Medicine, University of Lisbon, Lisbon, Portugal; Ohio State University

**Keywords:** β-glucan, *Bacteroides ovatus*, carbohydrate microarrays, polysaccharide utilization loci, protein-carbohydrate interactions, SusD-like proteins, X-ray crystallography

## Abstract

A multigene polysaccharide utilization locus (PUL) encoding enzymes and surface carbohydrate (glycan)-binding proteins (SGBPs) was recently identified in prominent members of *Bacteroidetes* in the human gut and characterized in Bacteroides ovatus. This PUL-encoded system specifically targets mixed-linkage β1,3-1,4-glucans, a group of diet-derived carbohydrates that promote a healthy microbiota and have potential as prebiotics. The BoSGBP_MLG_-A protein encoded by the *BACOVA_2743* gene is a SusD-like protein that plays a key role in the PUL’s specificity and functionality. Here, we perform a detailed analysis of the molecular determinants underlying carbohydrate binding by BoSGBP_MLG_-A, combining carbohydrate microarray technology with quantitative affinity studies and a high-resolution X-ray crystallography structure of the complex of BoSGBP_MLG_-A with a β1,3-1,4-nonasaccharide. We demonstrate its unique binding specificity toward β1,3-1,4-gluco-oligosaccharides, with increasing binding affinities up to the octasaccharide and dependency on the number and position of β1,3 linkages. The interaction is defined by a 41-Å-long extended binding site that accommodates the oligosaccharide in a mode distinct from that of previously described bacterial β1,3-1,4-glucan-binding proteins. In addition to the shape complementarity mediated by CH-π interactions, a complex hydrogen bonding network complemented by a high number of key ordered water molecules establishes additional specific interactions with the oligosaccharide. These support the twisted conformation of the β-glucan backbone imposed by the β1,3 linkages and explain the dependency on the oligosaccharide chain length. We propose that the specificity of the PUL conferred by BoSGBP_MLG_-A to import long β1,3-1,4-glucan oligosaccharides to the bacterial periplasm allows *Bacteroidetes* to outcompete bacteria that lack this PUL for utilization of β1,3-1,4-glucans.

**IMPORTANCE** With the knowledge of bacterial gene systems encoding proteins that target dietary carbohydrates as a source of nutrients and their importance for human health, major efforts are being made to understand carbohydrate recognition by various commensal bacteria. Here, we describe an integrative strategy that combines carbohydrate microarray technology with structural studies to further elucidate the molecular determinants of carbohydrate recognition by BoSGBP_MLG_-A, a key protein expressed at the surface of Bacteroides ovatus for utilization of mixed-linkage β1,3-1,4-glucans. We have mapped at high resolution interactions that occur at the binding site of BoSGBP_MLG_-A and provide evidence for the role of key water-mediated interactions for fine specificity and affinity. Understanding at the molecular level how commensal bacteria, such as prominent members of *Bacteroidetes*, can differentially utilize dietary carbohydrates with potential prebiotic activities will shed light on possible ways to modulate the microbiome to promote human health.

## INTRODUCTION

Throughout evolution, the human gut microbiota has evolved to efficiently target and degrade complex carbohydrate molecules derived from the human diet, popularly termed “dietary fiber.” These carbohydrates evade a complete metabolization by the human digestive system, which is intrinsically poor in complex carbohydrate active enzymes (CAZymes) (1, 2). As such, the microbial community complements the metabolic capacity of the human organism, producing metabolites that influence nutrition and health (3). Thus, changes in the carbohydrate influx will not only shape the microbiota composition and homeostasis but also have an impact on human physiology. Clarifying the molecular mechanisms that underlie this cross-communication is key to personalized medicine solutions and to fine-tuning therapies for diseases associated with a dysbiosis of the microbiota, including obesity and inflammatory bowel disease (4–6).

**FIG 1:**
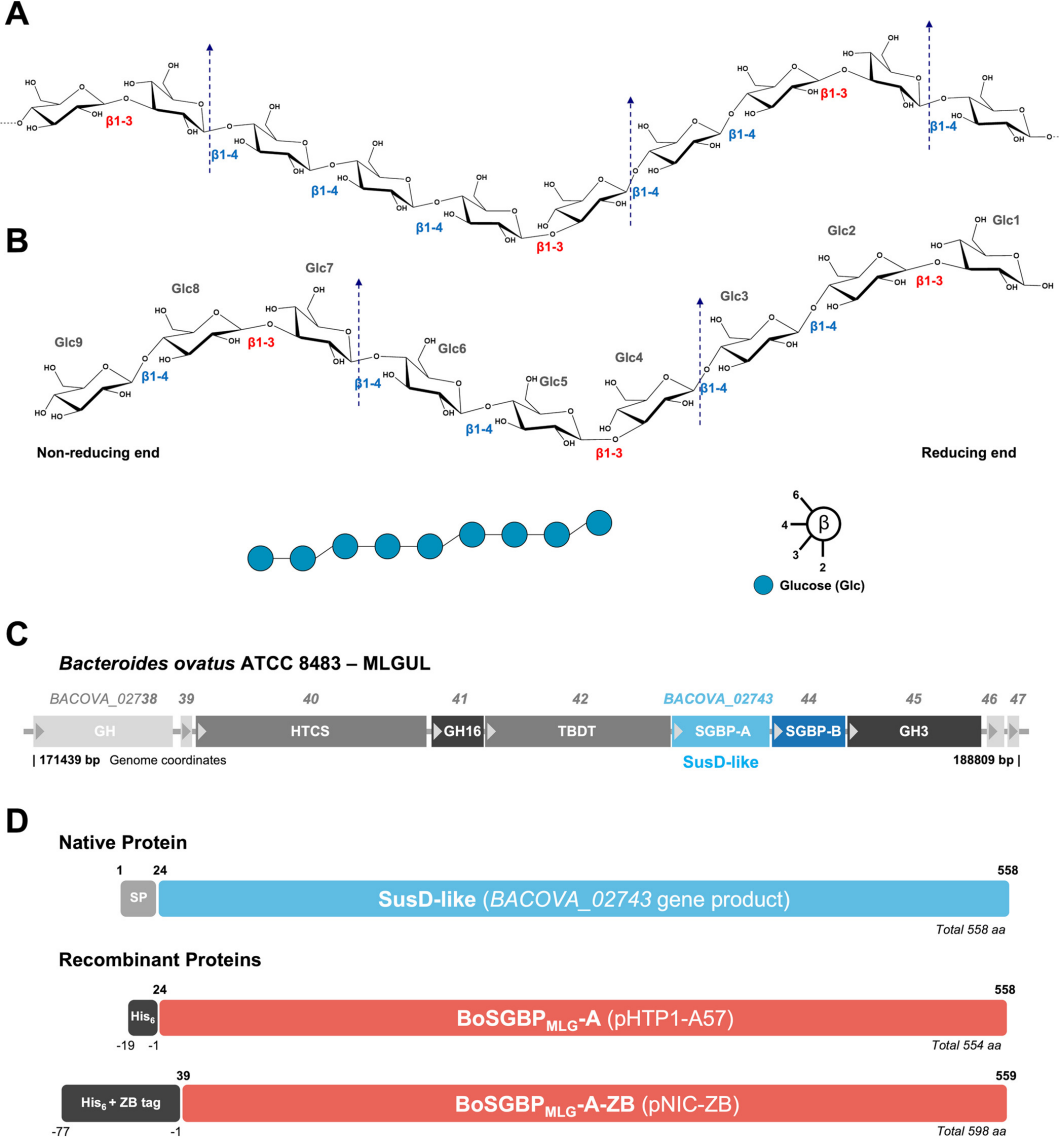
Mixed-linkage β1,3-1,4-glucans and Bacteroides ovatus polysaccharide utilization locus (PUL) targeting these carbohydrates, designated MLGULs (14). (A) Representative repeating units of the chemical structure of β1,3-1,4-glucans, which consist of a linear chain of β1,4-linked cellotriosyl or cellotetraosyl units spaced by β1,3-linked glycosidic linkages. Arrows indicate the specific sites of hydrolysis by the surface endoglucanase GH16 (BO2741) of the MLGUL. (B) Chemical structure and symbol nomenclature of the nonasaccharide (DP9) derived from barley β-glucan used in this study. Glucose units (Glc) are numbered from the reducing to the nonreducing end. Monosaccharide symbol representation follows the symbol nomenclature for glycans (SNFG) (52). (C) MLGUL system from B. ovatus strain ATCC 8483. (D) Molecular architecture of SusD-like protein BoSGBP_MLG_-A and diagram of the recombinant versions used in this study. Amino acid notation follows the numbering of the native protein, from N to C terminus. Gene or recombinant vector is in parentheses. SP, signal peptide; His_6_, recombinant tag with six histidine residues; ZB tag, recombinant Z_basic_ tag; aa, amino acids.

The symbiotic bacterium Bacteroides ovatus is a specialist in complex carbohydrates, carrying in its genome different colocalized gene clusters termed polysaccharide utilization loci (PUL). These encode CAZymes, surface carbohydrate (glycan)-binding proteins (generally designated SGBPs), TonB-dependent transporters (TBDTs), and transcriptional regulators, comprising complete systems to target and degrade major diet-derived and plant cell wall polysaccharides (1). An important group of complex carbohydrates with proven health benefits (7, 8) are the mixed-linkage β1,3-1,4-glucans, which are abundant in the endosperm of cereal grains of barley and oats and in algae and edible lichen (e.g., Icelandic moss) (9) and, more recently, have also been identified in microalgae (10). The β1,3-1,4-glucans are linear homopolysaccharides of d-glucopyranose constituted of blocks of three or four consecutive β1,4-linked residues (cellotriosyl or cellotetraosyl units, respectively) separated by single β1,3 linkages (Fig. 1A). The ratios of cellotriosyl and cellotetraosyl units differ with the source of the polysaccharide, resulting in different physicochemical properties (11). A β1,4-glucose-linked chain, as in the cellulose polysaccharide, is rigid and renders the polysaccharide less soluble in water, whereas the β1,3-linked glycosidic linkages confer flexibility and water solubility, creating kinks in the main chain and imposing a twisted conformation on the polysaccharide that challenges microbial degradation (9, 12).

In a pivotal study, Martens and colleagues identified a B. ovatus PUL (PUL 51) that is transcriptionally upregulated during growth on a mixed-linkage β-glucan from barley (Fig. 1B) (13). Recently, Tamura and colleagues demonstrated that copies of this locus, which they named mixed-linkage glucan utilization locus (MLGUL), are present in other *Bacteroidetes* species that are ubiquitous in the gut of human populations, pointing to the importance of the catabolism of β-glucans with mixed linkages by the microbiome (14). Structural and biochemical studies of two MLGUL CAZymes (a surface GH16 endo-β-glucanase and a periplasmic GH3 exo-β-glucosidase) and two SGBPs (BoSGBP_MLG_-A and BoSGBP_MLG_-B) have contributed to the characterization of the specificity of the MLGUL for mixed-linkage β1,3-1,4-glucans (14, 15). These studies provided evidence for a concerted model for polysaccharide enzymatic degradation and oligosaccharide targeting at the cell surface for transport via a SusC-like TBDT to enable complete saccharification to glucose in the periplasm. The SGBP protein encoded by the *BACOVA_02743* gene is a SusD homolog and was named BoSGBP_MLG_-A by Tamura and colleagues (15). In that study, the authors showed that the unique specificity of BoSGBP_MLG_-A toward mixed-linkage glucans is mediated by shape complementarity of its extended binding site with the twisted conformation of the oligosaccharide backbone and that the binding is dependent on the chain length up to the heptasaccharide, comprising two cellotriosyl repeats. That study also demonstrated the direct involvement of BoSGBP_MLG_-A in the functionality of the PUL, as mutations on the binding site and a gene knockout mutation blocked mixed-linkage β-glucan utilization by B. ovatus (15). Therefore, a detailed understanding of carbohydrate recognition by BoSGBP_MLG_-A is pivotal to understanding at the molecular level the utilization of β1,3-1,4-glucans by this symbiont in the microbiota.

The knowledge about carbohydrate recognition by proteins has been revolutionized by the advent of carbohydrate microarrays (16–19). This technology addresses the need for high-throughput methods to identify carbohydrate ligands for proteins and assign the specificities of carbohydrate-binding proteins. Having realized the importance of glucan recognition across all domains of life, we developed a “glucome” microarray as a screening tool for the study of glucan binding by proteins; the microarray comprises sequence-defined gluco-oligosaccharides with linear and branched sequences of different chain lengths and different linkages (20). We demonstrated its application to a wide range of glucan-binding proteins, including bacterial carbohydrate-binding modules (CBMs), anticarbohydrate antibodies, and immune lectins (20, 21).

Here, we report the carbohydrate microarray analysis of BoSGBP_MLG_-A to evaluate its binding to a wide range of carbohydrate structures and to assign the fine specificity toward β1,3-1,4-gluco-oligosaccharide sequences. We provide further evidence supporting that the binding pattern of BoSGBP_MLG_-A is different from those of other β1,3-1,4-glucan binding proteins and that BoSGBP_MLG_-A is unique in its requirement of and preferential binding to longer oligosaccharide chain lengths. By integrating the carbohydrate microarray data with data from affinity studies using microscale thermophoresis, we demonstrate the preferential binding of BoSGBP_MLG_-A to β1,3-1,4-gluco-oligosaccharide chains longer than the heptasaccharide reported previously by Tamura and colleagues (15). The crystal structure of BoSGBP_MLG_-A in complex with a β1,3-1,4-gluco-nonasaccharide solved at 1.45 Å reveals unique structural features that, combined with the results of isothermal titration calorimetry (ITC) and site-directed mutagenesis, enable us to assign at high resolution the molecular determinants of the carbohydrate binding and dependency on the chain length. We discuss the potential implications of the preferential binding of BoSGBP_MLG_-A to long β1,3-1,4-gluco-oligosaccharides for the PUL system’s functionality.

## RESULTS

### BoSGBP_MLG_-A targets mixed-linkage β1,3-1,4-glucans from different sources.

**FIG 2:**
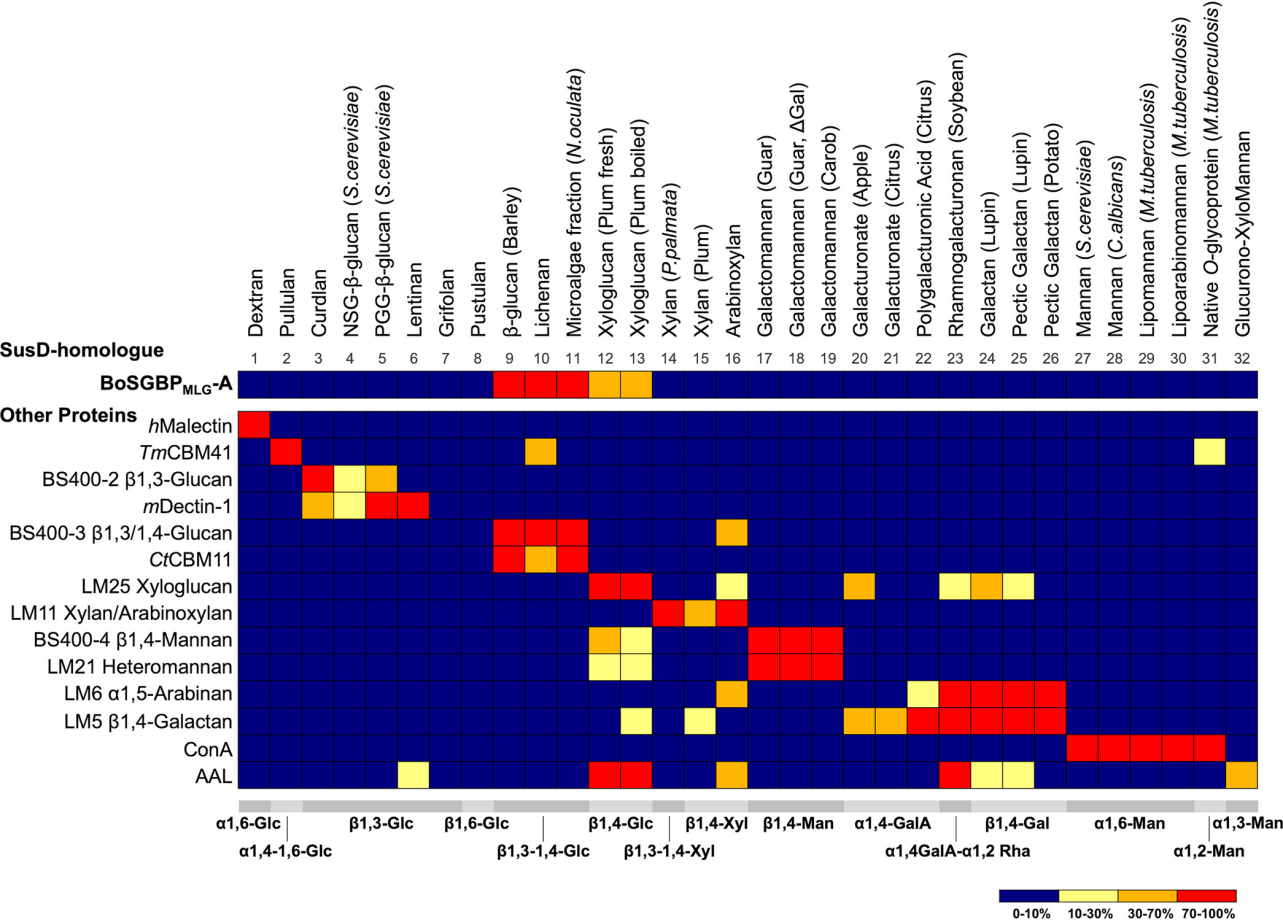
Carbohydrate binding by BoSGBP_MLG_-A using a structurally diverse saccharide microarray. Heatmap comparing the binding patterns of BoSGBP_MLG_-A and selected control proteins. The microarray comprised soluble saccharides of different origins (fungal, bacterial, plant, and microalgal polysaccharides or glycoproteins) (Table S1); the major backbone sequences are depicted at the bottom. The heatmap represents the relative binding intensities calculated as the percentage of the fluorescence signal intensity at 150 pg (0.5 mg/ml)/spot given by the saccharide probe most strongly bound by each protein (normalized as 100%). Results are detailed in Table S2. S. cerevisiae, Saccharomyces cerevisiae; N. oculata, Nanochloropsis oculata; P. palmata, Palmaria palmata; C. albicans, Candida albicans; M. tuberculosis, Mycobacterium tuberculosis; *h*Malectin, human malectin; *Tm*CBM41, CBM41 of Thermotoga maritima; *m*Dectin-1, murine dectin-1; *Ct*CBM11, CBM11 of Clostridium thermocellum; ConA, concanavalin A; AAL, Aleuria aurantia lectin.

The carbohydrate-binding properties of recombinant BoSGBP_MLG_-A (Fig. 1C) were first investigated using a microarray comprising carbohydrate probes (polysaccharides and glycoproteins) derived from fungi, bacteria, microalgae, and plants. These probes covered carbohydrate structural diversity with different glycosidic linkages in α or β configuration and were grouped by major backbone type as shown in Fig. 2 and in Table S1 in the supplemental material. Monoclonal antibodies (MAbs), carbohydrate-binding modules (CBMs), and lectins were also analyzed and showed binding profiles in accord with their reported carbohydrate-binding properties (Fig. 2 and Tables S2 and S3), thus validating the constructed microarray.

BoSGBP_MLG_-A showed strong binding to barley and lichenan β-glucans and to the β1,3-1,4-glucan-enriched fraction isolated from Nanochloropsis oculata microalgae, similar to the binding profiles of CBM11 of Clostridium thermocellum (*Ct*CBM11) and the β1,3-1,4-glucan-specific MAb BS400-3 (Fig. 2 and Table S2). The fact that BoSGBP_MLG_-A could bind mixed-linkage β1,3-1,4-glucans from different sources reflects the flexibility to accommodate the linear backbone with different ratios of β1,4-linked cellotriose to cellotetraose units spaced by β1,3-linkages (Fig. 1) (11). In accord with previous affinity data (15), BoSGBP_MLG_-A also interacted with branched xyloglucan fractions composed of a β1,4-linked glucose backbone, but with lower binding intensity. No interaction was observed with β1,3-glucans or with polysaccharides with a β1,4-linked backbone other than glucose, such as xylan and mannan, or to any of the other carbohydrate probes featured on the microarray, highlighting the preference of BoSGBP_MLG_-A for binding to mixed-linkage β1,3-1,4-glucans.

### BoSGBP_MLG_-A shows carbohydrate-binding specificity restricted to β1,3-1,4-gluco-oligosaccharides.

**FIG 3:**
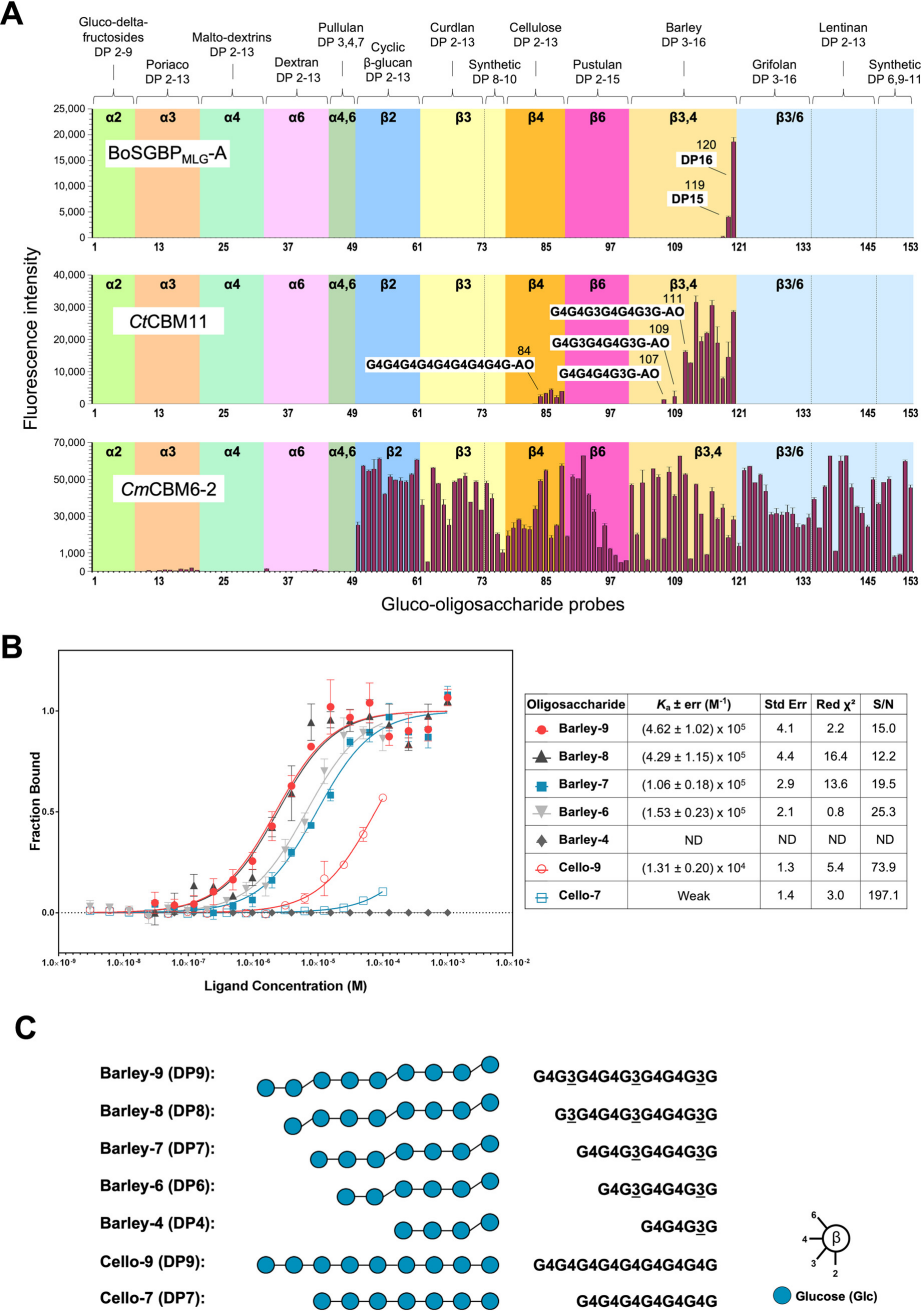
Determination of BoSGBP_MLG_-A oligosaccharide binding specificity and chain length requirements using sequence-defined oligosaccharides. (A) “Glucome” microarray comprising 153 sequence-defined gluco-oligosaccharides prepared as neoglycolipid (NGL) probes. *Ct*CBM11 (CBM11 of Clostridium thermocellum) and *Cm*CBM6-2 (CBM6-2 of Cellvibrio mixtus) were used as control proteins. The degree of polymerization (DP) and glucose linkages are indicated at the top of the panels. Some relevant carbohydrate probe sequences for binding to *Ct*CBM11 are depicted in panel B. G, glucose; AO, NGLs were prepared from reducing oligosaccharides by oxime ligation with an aminooxy (AO)-functionalized lipid (39). The binding signals are depicted as mean values of fluorescence intensities of duplicate spots for each probe arrayed at 5 fmol/spot (with error bars) and are representative of at least two independent experiments (details are in Table S4). (B) Microscale thermophoresis analysis of the interaction of BoSGBP_MLG_-A with sequence-defined gluco-oligosaccharides. Dose-response curves were fitted to a one-site binding model to obtain *K_a_* values. Error bars indicate the standard deviations from triplicate experiments (*n* = 3). Quality of the fitting is given by the standard error of regression and the reduced chi-square (Red χ^2^) parameters. S/N, signal-to-noise ratio. (C) Sequences of the gluco-oligosaccharides depicted from the nonreducing to the reducing end. Monosaccharide symbol representations follow the symbol nomenclature for glycans (SNFG) (52). The 3-linkages are underscored.

To investigate the binding specificity of BoSGBP_MLG_-A at the oligosaccharide level and the influence of oligosaccharide chain length, a structurally diverse gluco-oligosaccharide microarray that represented the major sequences found on glucans was used (Fig. 3A and Table S4) (20). The microarray was comprised of 153 sequence-defined gluco-oligosaccharides of different degrees of polymerization (DP2 to DP16; the degree of polymerization [DP] is the number of monomer units in a oligosaccharide), with linear and branched-chain lengths and homo- and mixed-linkages in α or β configurations prepared as neoglycolipid (NGL) probes (Table S4).

The microarray analysis revealed a restricted binding pattern of BoSGBP_MLG_-A, showing highly specific binding to gluco-oligosaccharide fractions with mixed β1,3-1,4 linkages derived from barley β-glucan (DP15 and DP16, probes number 119 and 120 in Fig. 3A and Table S4). Under the conditions of the analysis, no binding to linear β1,4-linked gluco-oligosaccharides up to DP13 was detected. While these results appear to contrast with the previously reported binding of BoSGBP_MLG_-A to β1,3-1,4-gluco-hexasaccharide and -heptasaccharide in solution using ITC (15), they might reflect the ability of the protein to access the required oligosaccharide sequence to bind productively in the microarrays. Supporting this were the different binding patterns displayed by the characterized glucan-binding *Ct*CBM11 and CBM6-2 of Cellvibrio mixtus (*Cm*CBM6-2) (Table S3). *Cm*CBM6-2 showed the predicted broad binding profile, recognizing all immobilized β-linked gluco-oligosaccharides (Fig. 3A and Table S4). Although presenting a β-glucan binding profile nearly as narrow as BoSGBP_MLG_-A, *Ct*CBM11 bound to barley-derived mixed β1,3-1,4-linked gluco-oligosaccharides with shorter chain lengths (e.g., DP7). As a type B CBM, *Ct*CBM11 recognizes the carbohydrate chain internally, which accounts for the requirement of a minimum chain length for access and recognition. *Ct*CBM11 targets, as a minimum binding motif, the mixed-linkage tetrasaccharide repeat with a β1,3 linkage at the reducing end, Glcβ1,4Glcβ1,4Glcβ1,3Glc (G4G4G3G) (22). In the microarrays, glycan probe presentation with the derivatization to the lipid via the reducing-end glucose prevented strong recognition of *Ct*CBM11 by probes with DP4 to DP6 (probes number 105, 107, and 109 in Table S4) by hindering access to the minimum-recognition binding motif. The same phenomenon may occur in the microarray analysis of BoSGBP_MLG_-A, but with a more pronounced effect, as it requires a longer chain length than the tetrasaccharide for binding (15), and since SusD-like proteins are larger than CBMs, it may therefore be more affected by steric hindrances in the microarray setup.

### Binding affinity of BoSGBP_MLG_-A is dependent on oligosaccharide chain length.

To further understand the chain length dependency of BoSGBP_MLG_-A and the influence of the β1,3-Glc linkage on the binding, microscale thermophoresis (MST) was used as a complementary technique to determine the affinities of the interactions of BoSGBP_MLG_-A with sequence-defined barley oligosaccharides of different DPs and increased numbers of β1,3-Glc linkages (Fig. 3B).

A series of purified longer-chain gluco-oligosaccharides (DP6 to DP9; barley-6 to -9) with mixed β1,3-1,4-linkages (Fig. 3C) were obtained from barley β-glucan after controlled enzymatic hydrolysis and characterized by negative-ion electrospray tandem mass spectrometry (MS/MS) (20) and nuclear magnetic resonance (NMR) (unpublished data). For comparison, β1,4-gluco-oligosaccharides (DP7 and DP9; cello-7 and -9) were prepared similarly by controlled acid hydrolysis of cellulose acetate and sequences assigned by MS.

The MST results also showed the preference of BoSGBP_MLG_-A for mixed β1,3-1,4-linked gluco-oligosaccharides (barley-) compared to β1,4-gluco-oligosaccharides (cello-) (Fig. 3B). The affinity for barley-9 (association constant [*K_a_*] of [4.62 ± 1.02] × 10^5^ M^−1^ [mean ± standard deviation]) was 1 order of magnitude higher than that for cello-9 (*K_a_* of 1.31 ± 0.20 × 10^4^ M^−1^). For the more weakly binding cello-7, it was not possible to determine the affinity with confidence, as under the conditions of the analysis, higher concentrations of the oligosaccharide could not be reached due to solubility issues. Importantly, the MST data demonstrated that the binding affinity was dependent both on the carbohydrate chain length and on the presence of β1,3 linkages along the chain (Fig. 3B and C). The interaction curves obtained with barley oligosaccharides showed a clear effect of the DP on increasing the affinity of the interaction, with barley-6/barley-7 and barley-8/barley-9 grouping with similar affinities. Considering their sequences, the increased affinity could be correlated with the increase in the number of β1,3-Glc linkages (Fig. 3C). These results suggest that BoSGBP_MLG_-A can bind to mixed-linkage β1,3-1,4-linked gluco-oligosaccharides with a minimum chain length of DP5/DP6 but prefers longer oligosaccharides containing at least three β1,3 linkages, e.g., DP8/DP9.

### The high-resolution structure of BoSGBP_MLG_-A in complex with a β1,3-1,4-gluco-nonasaccharide provides atomic detail on the long-chain interaction.

**FIG 4:**
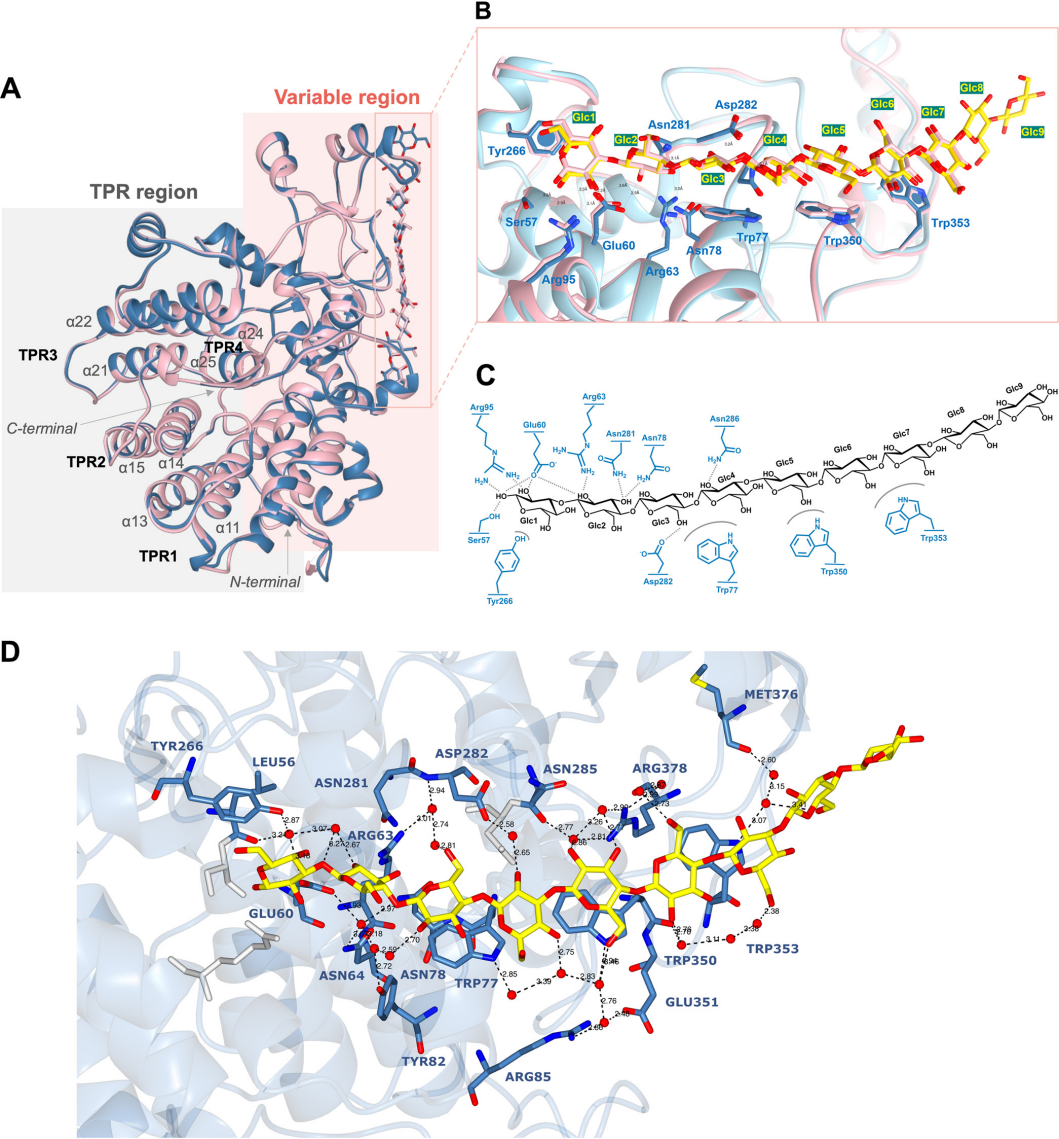
Three-dimensional structure of BoSGBP_MLG_-A in complex with barley-9 (G4G3G4G4G3G4G4G3G) at 1.43-Å resolution. (A) Cartoon representation of the overall canonical BoSGBP_MLG_-A SusD structure (in blue ribbon) showing two distinct regions: tetratricopeptide repeats (TPR) with four helix-turn-helix pairs, and a variable region with the bound barley-9 (G4G3G4G4G3G4G4G3G, in stick representation with blue carbon atoms and red oxygen atoms). The BoSGBP_MLG_-A–barley-9 complex structure is superposed with the structure of BoSGBP_MLG_-A (pink ribbon) in complex with barley-7 (G4G4G3G4G4G3G, in pink stick representation) (PDB ID 6E61) (15). (B) Close-up view of the binding site of BoSGBP_MLG_-A showing an overall match of sugar units 1 to 7. In both complexes, the three tryptophan residues form a platform that accommodates the carbohydrate twisted chain. The side chains of the protein residues involved in binding are shown as a stick model and labeled according to the respective model. Carbon atoms in barley-7 are colored in pink and barley-9 in yellow, nitrogen atoms are colored in dark blue, and oxygen atoms in red. Amino acids are numbered according to the native protein. Glucose units (Glc) are numbered from the reducing to the nonreducing end. (C) Schematic representation of the direct interactions between BoSGBP_MLG_-A and barley-9: curved surfaces, CH-π interactions; dashed lines, hydrogen bonding. (D) Ordered water network that extended intermolecular hydrogen bonding with different amino acids along the binding site and supported interaction with the entire oligosaccharide ligand (colored yellow). Contact distances are shown in Å and marked as dashed lines. The complete list of contacts is in Table S5.

To fully understand the molecular determinants of BoSGBP_MLG_-A’s unique specificity and chain length dependency, the three-dimensional (3-D) structure of BoSGBP_MLG_-A in complex with barley-9 (G4G3G4G4G3G4G4G3G) (Fig. 1B) was solved by X-ray crystallography to a resolution of 1.43 Å (Fig. 4 and Fig. S1). Data collection, processing, and refinement statistics are summarized in Table 1. Superposition with the structure of BoSGBP_MLG_-A in complex with barley-7 (G4G4G3G4G4G3G) (15) revealed an overall fold conservation (∼0.3-Å root mean square deviation [RMSD] between 516 Cα atom pairs) and an overall match with monosaccharide residues Glc1 to Glc7 (Fig. 4A and B). Most strikingly, in the complex with barley-9, two protein monomers were found in the asymmetric unit forming a protein-sugar-sugar-protein supramolecular assembly, where the protein interacts with a dimerized carbohydrate consisting of two barley-9 chains related by the 2-fold noncrystallographic axis (Fig. S2). That is, one complete chain of barley-9 could be modeled flanked on one side by one protein molecule (chain A) and on the opposite side by a second antiparallel barley-9 molecule that is flanked by the second protein monomer (chain B). The antiparallel barley9A-barley9B dimer is promoted by 4 direct symmetrical hydrogen bonding contacts between pairs Glc3A(C6-OH)-(OH-C2)Glc8B, Glc5A(C2-OH)-(OH-C6)Glc6B, Glc6A(C6-OH)-(OH-C2)Glc5B, and Glc8A(C2-OH)-(OH-C6)Glc3B. Besides these direct contacts, sugar oligomerization is maintained by 8 water-mediated contacts established between glucose pairs Glc2A-Glc9B, Glc3A-Glc7B, Glc4A-Glc7B, Glc4A-Glc6B, Glc5A-Glc5B, Glc6A-Glc4B, Glc7A-Glc4B, and Glc9A-Glc2B. For clarity in Fig. 4, only chain A is represented and discussed, but all observations were systematically cross-checked in both chains A and B. The high-resolution data (1.43 Å) and the presence of positive peaks of residual electron density in the *mF*_obs_ − *DF*_calc_ map indicate that alternate conformations are possible for the C6-OH groups of Glc3, Glc5, and Glc6 in chain B, but, overall, the same protein-carbohydrate contacts were maintained compared to chain A component molecules.

**TABLE 1:** X-ray diffraction data collection and 3-D structure refinement statistics

Parameter	Value(s) for BoSGBP_MLG_-A-ZB–barley-DP9^*a*^
Data collection and processing	
Beamline	I04 (Diamond Light Source)
Wavelength (Å)	0.9795
Space group	*P* 2_1_ 2_1_ 2_1_
Unit cell parameters	
* * *a, b, c* (Å)	87.84, 88.44, 156.15
* * α, β, γ (°)	90, 90, 90
Resolution range (Å)	62.32–1.43 (1.55–1.43)
Solvent content (%)	45
Protein molecules in the asymmetric unit	2
Matthews coefficient (Å^3^ · Da^−1^)	2.24
* I*/σ (*I*)	1.6 (19.1)
Wilson B factor (Å^2^)	13.1
* R*_merge_ ^*b*^	0.05 (0.78)
* R*_p.i.m._ ^*c*^	0.02 (0.35)
CC_1/2_^*d*^	1.00 (0.74)
Multiplicity	9.0 (5.6)
Total no. of reflections	1,569,176 (48,717)
No. of unique reflections	175,289 (8,764)
Completeness (%)	96.5 (69.0)

Refinement statistics	
Resolution range (Å)	44.86–1.43
No. of:	
Protein atoms	8,337
Carbohydrate atoms	216
Water molecules	974
Other solvent atoms	168
* R*_work_ ^*e*^	0.1638
* R*_free_ ^*f*^	0.1897
RMSD	
Bond length (Å)	0.010
Bond angle (°)	1.140
Avg B factor (Å^2^) of^*g*^:	
Main chain (A, B)	18.39, 21.47
Side chain (A, B)	21.50, 24.38
G4G3G4G4G3G4G4G3G (A, B)	35.95, 35.00
Magnesium ions (A, B)	16.94, 16.33
Sodium ions	25.83
TAM	56.83
Azide ions	42.92
GOL	35.5
PEG	49.04
Water molecules (974)	31.10
Ramachandran statistics (%)	
Favored	97.78
Allowed	2.22
Outliers	0.00

a Values for the outer shell are given in parentheses. The data have been deposited in the Protein Data Bank under PDB ID 7NOX.

b $R_{\text{merge}}=\left[\sum_{\mathrm{hkl}}\sum_{i=1}^{n}\left|I_{i}\left(\mathrm{hkl}\right)-\bar{I}\left(\mathrm{hkl}\right)\right|\right]/\left[\sum_{\mathrm{hkl}}\sum_{i=1}^{n}I_{i}\left(\mathrm{hkl}\right)\right]$, where I is the observed intensity and $\bar{I}$ is the statistically weighted average intensity of multiple observations.

c $R_{\text{p}.\text{i}.\text{m}.}=\left[\sum_{\mathrm{hkl}}\sqrt{1/\left(n-1\right)}\sum_{i=1}^{n}\left|I_{i}\left(\mathrm{hkl}\right)-\bar{I}\left(\mathrm{hkl}\right)\right|\right]/\left[\sum_{\mathrm{hkl}}\sum_{i=1}^{n}I_{i}\left(\mathrm{hkl}\right)\right]$, a redundancy-independent version of $R_{\text{merge}}$.

d CC_1/2_, correlation coefficient (CC) between intensities from random half-datasets.

e $R_{\text{work}}=\left[\sum_{\mathrm{hkl}}\left|\left|F_{\text{obs}}\left(\mathrm{hkl}\right)\right|-\left|F_{\text{calc}}\left(\mathrm{hkl}\right)\right|\right|\right]/\left[\sum_{\mathrm{hkl}}\left|F_{\text{obs}}\left(\mathrm{hkl}\right)\right|\right]$, where $\left|F_{\text{calc}}\right|$ and $\left|F_{\text{obs}}\right|$ are the calculated and observed structure factor amplitudes, respectively.

f *R*_free_ is calculated for a randomly chosen 5% of the reflections.

g TAM, Tris(hydroxyethyl)aminomethane; GOL, glycerol; PEG, polyethylene glycol; (A, B), indicates the corresponding values for chain A and chain B, respectively, in the asymmetric unit.

### Molecular determinants of the carbohydrate-binding specificity are supported by a complex water-mediated hydrogen bonding network.

The structure of the BoSGBP_MLG_-A–barley-9 complex showed an ∼41-Å-long binding platform that accommodated and accompanied the natural bends and kinks of this mixed-linkage oligosaccharide (Fig. 4B and C). All nine glucose residues of the ligand could be modeled in the electron density in the lowest-energy ^4^C_1_ chair conformation. BoSGBP_MLG_-A bound to the barley-9 through the oligosaccharide-reducing end (Glc1), and multiple protein contacts up to Glc8 stabilized the interaction. These included CH-π stacking, direct hydrogen bonding, and an extensive water-mediated network (Fig. 4C and D and Table S5). The nonreducing terminal Glc9 residue showed no interaction with any amino acid, directly or indirectly.

**FIG 5:**
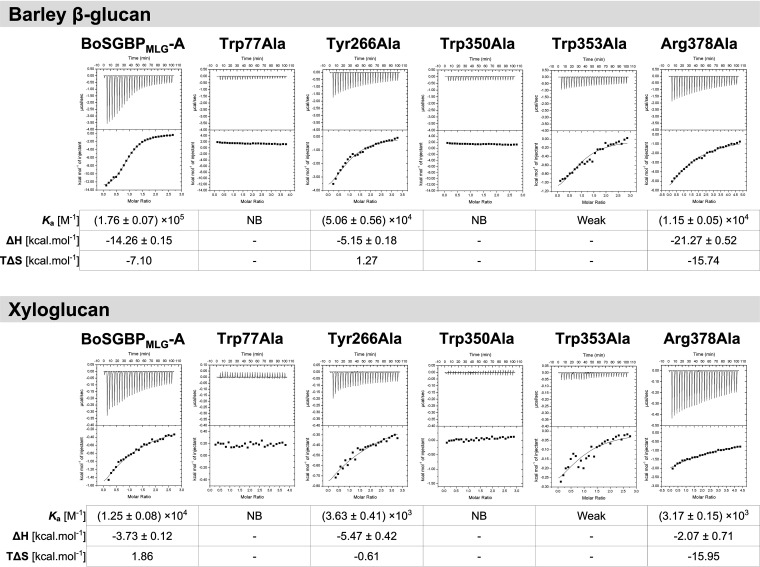
Interactions of BoSGBP_MLG_-A and its site-directed mutants with barley β-glucan and xyloglucan polysaccharides measured using isothermal titration calorimetry (ITC). Titrations were conducted at 25°C in 50 mM HEPES buffer (pH 7.5) with 100 mM NaCl, 5 mM CaCl_2_, and 5 mM TCEP. The top half of each panel shows the raw heats of binding, whereas the bottom shows the integrated heats of dilution-corrected data. The curves represent the best fits to a single-site binding model. NB, no binding observed; weak, value was too low to be quantified.

As in the BoSGBP_MLG_-A structure with the heptasaccharide (15), the triad of residues formed by Trp77, Trp350, and Trp353 constitutes the center of the platform, interacting with the internal sequence of the gluco-oligosaccharide chain (Glc3-Glc7) (Fig. 4C). The CH-π stacking established between the aromatic side chains of the triad and the saccharide rings is the main force positioning the ligand in the binding site. This is mainly mediated by Trp77 and Trp350, as mutating any of these residues to alanine abolished the binding to barley β-glucan and xyloglucan, as determined by ITC analysis (Fig. 5), corroborating the reported results using affinity gel electrophoresis (15). The torsion in the Trp353 side chain in relation to Trp77 and Trp350 causes a curvature of the binding site that optimizes the CH-π interactions with Glc6 and Glc7 and best accommodates the natural bends imposed by the mixed β1,3-1,4 linkages of the oligosaccharide. The importance of these Trp353-mediated interactions for the affinity of BoSGBP_MLG_-A was corroborated by the unquantifiable weak interaction observed with the Trp353Ala mutant (bearing a change from Trp to Ala at position 353) (Fig. 5). At the reducing end, the aromatic ring of Tyr266 establishes a CH-π stacking with Glc1, and mutation of this residue caused a 3.5-fold decrease in the *K_a_* value for both barley β-glucan and xyloglucan polysaccharides, which corroborates the importance of this interaction for anchoring and orienting the oligosaccharide. In addition, the direct hydrogen bonding contacts and electrostatic interactions promoted by eight amino acid residues with the reducing-end tetrasaccharide stretch (Glc1 to Glc4) (Fig. 4C and Table S5) are conserved in the structures of both BoSGBP_MLG_-A–oligosaccharide complexes.

The high resolution of the BoSGBP_MLG_-A–barley-9 structure enabled us to identify 21 ordered water molecules constituting an extensive and complex water network that mediates indirect hydrogen bonding contacts with 18 amino acid residues, supporting the entire oligosaccharide from Glc1 to Glc8 (Fig. 4D and Table S5). In particular, four water molecules were shown to mediate interactions of Arg378 Nη1 and Nη2 with O2 and O3 of Glc5 and O6 of Glc6. Also, Arg378 Nη1 hydrogen binds to the Oγ of Ser349, which in turn is water bridged to Asn286, a direct contact of Glc4. The mutation of Arg378 to Ala resulted in a decrease of affinity by approximately 15-fold for barley β-glucan polysaccharide but only 3.9 times for xyloglucan, evidencing the importance of these water-mediated contacts for interaction with the mixed-linkage glucans (Fig. 5). At the nonreducing end, a water-mediated triad was also identified between the carbonyl of the main chain of Met376 and Trp353, mediating the interaction with the Glc8 residue (Fig. 4D). Two additional water molecules promoted the contact between the O6 of Glc8, the Nε1 from Trp353, and the Met376 carbonyl oxygen. These water-mediated contacts constitute the protein-ligand interactions holding Glc8 and explain the limit for chain length recognition by BoSGBP_MLG_-A.

## DISCUSSION

The recent characterization of a Bacteroides ovatus PUL targeting complex dietary β1,3-β1,4-glucans provided molecular insight into the specificity of surface carbohydrate binding proteins toward these mixed-linkage polysaccharides and demonstrated the crucial role of substrate binding by the BoSGBP_MLG_-A SusD homologue in the functionality of the PUL (15). In particular, the X-ray crystallography structure of BoSGBP_MLG_-A SusD in complex with a barley-derived heptasaccharide (G4G4G3G4G4G3G) revealed that the first level of specificity is determined by shape complementarity of the binding site with the twisted conformation of the glucan chain with mixed β1,3-1,4 linkages (15). Here, we combine the power of the carbohydrate microarray technology with quantitative affinity studies and high-resolution structure determination of BoSGBP_MLG_-A in complex with a higher-affinity oligosaccharide ligand that we have identified. We visualize thereby details of the molecular determinants underlying the unique specificity of BoSGBP_MLG_-A toward glucans with mixed β1,3-β1,4 linkages.

We demonstrate that the binding specificity of BoSGBP_MLG_-A toward β1,3-1,4-gluco-oligosaccharide sequences differs from those of other β-glucan binding proteins (e.g., *Ct*CBM11 and *Cm*CBM6-2), with the binding affinity being dependent on the chain length and on the number and position of β1,3 linkages. While corroborating that BoSGBP_MLG_-A is highly specific toward mixed-linkage β1,3-1,4-gluco-oligosaccharides, with a minimum chain length of DP5/DP6 (15), we demonstrate that the affinity increases with the addition of a β1,3 linkage after every three glucose units. The crystal structure of the BoSGBP_MLG_-A–barley-9 (G4G3G4G4G3G4G4G3G) complex reveals an ∼41-Å-long binding site (compared to the 36-Å-long binding site with the heptasaccharide) comprising an extended platform that accommodates the oligosaccharide through its reducing end up to Glc8. As observed in the structure of BoSGBP_MLG_-A complexed with the heptasaccharide (15), a triad of aromatic amino acids (Trp77, Trp350, and Trp353) form a CH-π stacking surface that accommodates the internal sequence of the oligosaccharide. These interactions dominate the specificity and set the minimum motif length by creating a curvature at the binding site into which the kink of a β1,3-glycosidic bond between Glc4 and Glc5 fits best. Mutating any of the triad residues to an alanine abrogates (Trp77 and Trp350) or significantly diminishes (Trp353) the affinity of the interaction. Thus, the minimum binding motif must contain at least 5 glucose residues and the sequence G3G4G4G3G.

The high-resolution data obtained for the BoSGBP_MLG_-A–barley-9 complex structure identified an ordered water network that promotes extended intermolecular hydrogen bonding that is essential to support the long oligosaccharide ligand. In particular, the side chain of Arg378 makes four water-mediated hydrogen bonds that likely contribute to the specificity by stabilizing the Glc4-Glc6 stretch that accommodates the minimum binding motif. The importance of this residue for the specificity is supported by the effect of the point mutation of Arg378 to an Ala, which reduced the affinity of BoSGBP_MLG_-A for barley β-glucan more than it reduced its affinity for xyloglucan and had an effect comparable to that of the mutation of Tyr266, which stabilizes the reducing end through a CH-π interaction. The importance of solvent organization to BoSGBP_MLG_-A’s affinity is also evidenced by the pair Trp353 and Met376, which sets the preference for longer oligosaccharides by bridging with two water molecules to interact with the Glc8 monosaccharide. The kink imposed on the oligosaccharide chain by the third β1,3-glycosidic linkage between Glc7 and Glc8 approximates Glc8 monosaccharide to the protein surface and likely promotes the interaction through these water-mediated contacts. Thus, the water-mediated interactions are tailoring the BoSGBP_MLG_-A binding site to mixed-linkage glucans and contributing to the specificity and increased affinity with oligosaccharide chain length. The presence of ordered water molecules is often observed in carbohydrate-protein crystal structures, allowing specific additional interactions to occur between the protein and the ligand (23). The importance of these water-mediated hydrogen bonding networks in modulating ligand affinity and specificity in protein-ligand interactions is increasingly being recognized, giving particular attention to the balance of enthalpic and entropic contributions to binding (23–26).

The direct hydrogen bonding/electrostatic interactions at the reducing end and additional water network interactions mediating carbohydrate binding by BoSGBP_MLG_-A will likely allow for flexibility of the binding site to accommodate structural differences of mixed-linkage glucans, i.e., the ratios of cellotriosyl (G4G4G) and cellotetraosyl (G4G4G4G) units spaced by β1,3 linkages, explaining the ability demonstrated here to recognize mixed β1,3-1,4-linked glucans from different sources.

The protein-sugar-sugar-protein assembly that constitutes the asymmetric unit of the BoSGBP_MLG_-A–barley 9 complex (Fig. S2) may be a consequence of a high local concentration of the oligosaccharide, which might be compensated by the formation of aggregates, helping the crystal nucleation event. The observed assembly in the crystal structure could also reflect a unique means for B. ovatus to entrap long oligosaccharides and prevent the formation of aggregates at high cell surface oligosaccharide concentrations, but experimental validation is required for biological relevance.

Our study and that of Tamura and colleagues (14, 15) raise the question as to how the preference of BoSGBP_MLG_-A for longer oligosaccharides impacts on the function of the PUL-encoded system that occurs widely in the *Bacteroidetes* species. The hypothesis is that BoSGBP_MLG_-A could confer specificity and preference for the import of long β1,3-1,4-glucan oligosaccharides generated by the endo-glucanase GH16 (*BACOVA_02741*) through the TBDT (*BACOVA_02742*). BoSGBP_MLG_-A would then likely be playing a role in the rapid import and scavenging of long oligosaccharides to the periplasm rather than targeting the polysaccharide to the surface, to enhance hydrolysis by the enzyme, as initially proposed for the function of these proteins (27). This would complement the action of the other SGBP, BoSGBP_MLG_-B (*BACOVA_02744*), which is suggested to assist in capturing oligosaccharides from the environment (15).

Recent studies demonstrate that SusD homologues of Bacteroides thetaiotaomicron can be intimately associated with the cognate TBDT SusC, forming a complex that works as a “pedal-bin” mechanism for import, with SusD functioning as a lid that opens and closes for carbohydrate capture and delivery to the transporter (28, 29). For MLGULs, the encoded import mechanism of mixed-linkage β1,3-1,4-gluco-oligosaccharides is yet to be elucidated. Nevertheless, the hypothesis raised would justify the evolution of BoSGBP_MLG_-A toward a higher affinity for longer oligosaccharides, as observed for other SusD homologues (29, 30). As endo-acting *Bacteroides* glycanases generate large oligosaccharides (31, 32), BoSGBP_MLG_-A could also be optimized to bind to major products generated locally by the PUL endo-glucanase. The preference for longer oligosaccharides would be energetically more favorable and would enable B. ovatus and other *Bacteroidetes* to outcompete bacteria that lack this PUL-encoded system.

## MATERIALS AND METHODS

### Plasmid construction and site-directed mutagenesis.

The molecular architectures and sequences of primers designed for each construct are displayed in Fig. 1D and Table S6. All constructs were truncated to exclude the signal peptide and N-terminal lipidation cysteine residue predicted with SignalP 4.1 and LipoP 1.0, respectively (33, 34). All resulting recombinant proteins have an N-terminal hexahistidine (His_6_) tag fusion for purification through ion metal affinity chromatography (IMAC) and immunodetection.

For carbohydrate-binding experiments (microarray, ITC, and MST analysis), the high-throughput cloning of the putative carbohydrate-binding SusD-like protein BoSGBP_MLG_-A (Fig. 1C) was performed following the established protocols of NZYTech Ltd. (Lisbon, Portugal). In brief, the gene *BACOVA_02743* encoding the SusD homologue (amino acid residues 24 to 558) was amplified by PCR from Bacteroides ovatus strain ATCC 8483 (NCBI:txid411476) genomic DNA, using specific primers. The pCR product was subsequently cloned into the pHTP1 expression vector using the NZYEasy cloning kit (NZYTech Ltd., Portugal) for ligation-independent cloning (LIC) technology. The pHTP1 vector contains a kanamycin resistance cassette for selection. In pHTP1-A57, the derivative is under the control of a T7 promoter.

For crystallization, BoSGBP_MLG_-A (residues 39 to 558) was recloned into the pNIC-ZB vector (Structural Genomics Consortium [SGC]; GenBank accession number GU452710), also using the LIC method and with the pHTP1-A57 DNA as the template, originating the BoSGBP_MLG_-A-ZB construct (Fig. 1C) (35). The pNIC-ZB complementary sequences were included in the forward and reverse primers for LIC. The vector was linearized with the restriction enzyme BsaI (Thermo Fisher Scientific). Both the linearized vector and the amplified insert were treated with T4 DNA polymerase (Thermo Fisher Scientific) to create the complementary overhangs, and they were annealed at a 1:2 molar ratio (vector/insert). For mutagenesis, site-directed mutants of BoSGBP_MLG_-A were created in the pHTP-A57 vector using the PCR-based NZYMutagenesis kit (NZYTech Ltd., Portugal) according to the manufacturer’s instructions. The fidelity of all plasmid constructs was verified by DNA sequencing (Stab Vida Lda, Portugal).

### Protein production and purification.

For carbohydrate microarray binding assays, the high-throughput expression and purification of BoSGBP_MLG_-A was done using the established protocols of NZYTech Ltd. (Lisbon, Portugal) (36). In brief, Escherichia coli strain BL21(DE3) harboring the plasmid pHTP1-A57 was cultured in NZY autoinduction Luria-Bertani (LB) medium (NZYTech Ltd., Portugal) supplemented with 50 μg/ml kanamycin at 37°C until reaching an optical density at 600 nm (OD_600_) of ≈1.5, with further overnight incubation at 25°C. Cultured cells were resuspended in NZY bacterial cell lysis buffer, and protein was purified by IMAC using the 96-well-plate vacuum manifold setting and eluted in 50 mM HEPES buffer, pH 7.5, containing 1 M NaCl, 5 mM CaCl_2_, and 300 mM imidazole.

For other experiments, E. coli BL21(DE3) cells harboring the plasmids encoding BoSGBP_MLG_-A, BoSGBP_MLG_-A-ZB, and mutants were cultured at 37°C to mid-exponential phase (OD_600_ = 0.6) in selective LB medium with kanamycin (50 μg/ml). Recombinant protein expression was induced by adding IPTG (isopropyl-β-d-thiogalactopyranoside) (1 mM), followed by further, overnight incubation at 19°C. Cells were harvested by centrifugation at 5,000 × *g* for 15 min at 4°C and lysed using sonication (0.5 cycle, 80% amplitude, MS7 probe) (UP100H ultrasonic processor; Hielscher Ultrasonics GmbH) in 50 mM HEPES, pH 7.5, 1 M NaCl, 10 mM imidazole, 5 mM CaCl_2_, and 10 mM β-mercaptoethanol buffer supplemented with a mini EDTA-free protease inhibitor cocktail tablet (Roche), DNase (1 μg/ml), MgCl_2_ (5 mM), and lysozyme (300 μg/ml). Cellular debris was removed by centrifugation at 13,000 × *g* for 30 min at 4°C. Filtrated supernatant (0.2-μm pore size) was loaded onto a HiTrap IMAC HP 5-ml column (GE Healthcare, Little Chalfont, Buckinghamshire, UK), and protein was eluted with an imidazole gradient concentration (10 to 500 mM) in the ÄKTA start chromatography system (GE Healthcare). Peak fractions were analyzed by SDS-PAGE (10% polyacrylamide Tris-tricine gels stained with Coomassie blue) using the broad-range protein marker NZYColour II (NZYTech, Ltd.). Fractions containing the purified proteins were buffer exchanged to 50 mM HEPES, pH 7.5, 100 mM NaCl, 5 mM CaCl_2_, and 5 mM TCEP [Tris(2-carboxyethyl)phosphine hydrochloride] using HiTrap desalting columns (GE Healthcare) in the ÄKTA start system. For crystallization assays, BoSGBP_MLG_-A-ZB was concentrated using a Vivaspin turbo 15 centrifugal concentrator (Sartorius, Goettingen, Germany) with a molecular-mass cutoff of 30 kDa (3,500 × *g*, 4°C). Purified proteins were quantified by UV-visible light spectrophotometry (OD_280_) with a SpectraDrop microvolume 24-microwell plate and SpectraMax 190 microplate reader (Molecular Devices).

### Polysaccharides, oligosaccharides, and carbohydrate-binding proteins.

The soluble polysaccharides used for ITC analysis (barley β-glucan and tamarind seed xyloglucan) were purchased from Megazyme (Bray, Ireland) and prepared according to the manufacturer’s instructions. The barley-4b oligosaccharide used in the MST analysis was purchased from Megazyme (O-BGTETB). The β1,4-oligosaccharides (cello-DP7 and -DP9) and the mixed-β1,3-1,4-linked gluco-oligosaccharides DP6 to DP9 used in the MST analysis were prepared by controlled acid hydrolysis of cellulose acetate (20) or by controlled enzymatic hydrolysis of barley β-glucan with lichenase (Megazyme), respectively. The resulting mixtures were fractioned by gel filtration chromatography with a Bio-Gel P4 column (Bio-Rad) and analyzed by mass spectrometry (20) and NMR (unpublished data).

The polysaccharides, glycoproteins, and oligosaccharides used in the microarray analysis are described in Tables S1 and S4. Selected carbohydrate-binding proteins with characterized specificity were analyzed as controls and comprised monoclonal antibodies, bacterial carbohydrate-binding modules (CBMs), and lectins. These are detailed in Table S3. All CBMs and human malectin were produced in E. coli as recombinant proteins with an N-terminal His_6_ tag, except for *Ct*CBM11 from Clostridium thermocellum, which carried a C-terminal His_6_ tag. *Ct*CBM11 (22) and human malectin (37) were prepared as previously described. *Cm*CBM6-2, from Cellvibrio mixtus, was kindly provided by Harry Gilbert (University of Newcastle, UK). *Tm*CBM41, from the marine hyperthermophile Thermotoga maritima, was kindly provided by Alisdair Boraston (University of Victoria, Canada).

### Carbohydrate microarray analysis.

Information on the saccharide and oligosaccharide probes, generation of the microarrays, imaging, and data analysis are described in the supplemental glycan microarray document (Table S7) based on MIRAGE (minimum information required for a glycomics experiment) guidelines (38).

For microarray-screening analysis, the following two types of microarrays were used: (i) a microarray designated “fungal, bacterial, microalgae, and plant saccharide microarray” featuring 32 saccharides (polysaccharides and glycoproteins) derived from fungi, bacteria, microalgae, and plants (Table S1), and (ii) a “gluco-oligosaccharide microarray,” comprising 153 sequence-defined gluco-oligosaccharides (Table S4). The oligosaccharides (up to 100 μg) were prepared as NGL probes by microscale conjugation via reducing-end glucose with the aminooxy (AO)-functionalized lipid 1,2-dihexadecyl-*sn*-glycero-3-phosphoethanolamine (AOPE) using oxime ligation in a solvent system, such as CHCl_3_/MeOH/H_2_O/AcOH, 25:25:8:1, analyzed by high-performance thin-layer chromatography (HPTLC) and mass spectrometry, and accurately quantified in solution as previously described (20, 39). For construction of the microarrays, the polysaccharides and glycoproteins (0.03 and 0.1 ng per spot) or the NGL probes (2 and 5 fmol/spot) in the form of liposomes were printed and immobilized noncovalently as duplicate spots on nitrocellulose-coated glass slides (UniSart 3D microarray slide; Sartorius, Goettingen, Germany), following established protocols (20, 40). The fluorescent dye cyanine 3 was included in the printing solution as a tracer for quality control of the arraying process and for localization of the printed spots.

The microarrays were probed with BoSGBP_MLG_-A following described protocols (20, 40). In brief, the nitrocellulose surface was blocked with 3% bovine serum albumin (BSA) (product number A8577; Sigma-Aldrich) in 5 mM HEPES buffer, pH 7.4, 150 mM NaCl supplemented with 5 mM CaCl_2_ (3% BSA in HBS-Ca), followed by incubation with the protein diluted in the binding buffer (1% BSA in HBS-Ca). For the saccharide microarrays, BoSGBP_MLG_-A was analyzed at 100 μg/ml, and binding was detected using an antipolyhistidine monoclonal antibody (Ab1) (product number H1029; Sigma-Aldrich) preincubated (for 15 min) with an biotinylated anti-mouse IgG (Ab2) (product number B7264; Sigma-Aldrich) at 10 μg/ml. For the gluco-oligosaccharide microarray, BoSGBP_MLG_-A precomplexed with Ab1 and Ab2 at a ratio of 1:2:2 (by weight) was analyzed at 20 μg/ml. The protein-antibody complexes were prepared by preincubating Ab1 and Ab2 for 15 min, followed by incubation with BoSGBP_MLG_-A for 15 min and final dilution in binding buffer for microarray overlay.

In parallel, the microarrays were analyzed with sequence-specific proteins for quality control and data validation (Table S3). The His-tagged murine dectin-1 and human malectin lectins were tested at 5 μg/ml, and the binding was detected with the precomplex of antibodies Ab1 and Ab2 at a final concentration of 10 μg/ml in the binding buffer. The His-tagged CBMs *Tm*CBM41, *Cm*CBM6-2, and *Ct*CBM11 were analyzed at final concentrations of 5 to 20 μg/ml, precomplexed with Ab1 and Ab2 at a ratio of 1:3:3 (by weight), and diluted in 1% BSA in HBS-Ca after blocking with 3% BSA in HBS-Ca. The monoclonal antibodies BS400-2, BS400-3, BS400-4, LM5, LM6, LM11, LM21, and LM25 were analyzed at 10 μg/ml using specific biotinylated secondary antibodies for detection. In brief, after blocking with 0.02% casein (catalog number 37583; Thermo Scientific), 1% BSA in HBS-Ca, the microarrays were probed with the antibodies prepared in the same buffer, followed by incubation with 3 μg/ml biotinylated anti-mouse IgG (product number B7264; Sigma-Aldrich), anti-rat IgG (product number B7139; Sigma-Aldrich), or anti-rat IgM (catalog number 612-4607; Rockland) as appropriate. The lectins Aleuria aurantia lectin (AAL) and concanavalin A ConA were analyzed at 2 and 5 μg/ml, respectively, using a single-step overlay protocol for biotin-tagged samples. In brief, the array was blocked with 3% BSA in HBS-Ca, followed by incubation with the different lectin solutions, prepared in the binding buffer.

For all the analyses, Alexa Fluor 647-labeled streptavidin (Molecular Probes, 1 μg/ml) was used for the fluorescence readout. Binding assays were conducted at room temperature. All microarray slides were scanned with the GenePix 4300A fluorescence scanner, and quantitation of the fluorescence was performed using GenePix Pro software (Molecular Devices). Microarray data were analyzed using a software developed by Mark Stoll from the Glycosciences Laboratory (Imperial College London, UK) (41). The parameters for recording the fluorescence images were selected considering the signal-to-noise ratio and the saturation of the signal in the different experiments. These are detailed in the MIRAGE (38) document in Table S7. The binding signals in the microarrays were dose dependent. The results given are plotted as the average values from two replicates for binding signals at 0.1 ng/spot (saccharides) or 5 fmol/spot (NGL).

### MST.

The affinities of the interactions between BoSGBP_MLG_-A and different mixed-linkage β1,3-1,4- and β1,4-gluco-oligosaccharides (barley- and cello-, respectively) were measured using microscale thermophoresis (MST) in a Monolith NT.115 instrument (NanoTemper Technologies, Germany). BoSGBP_MLG_-A was labeled with the red fluorescent dye NT-647 using the Monolith NT protein-labeling kit red-NHS (*N*-hydroxysuccinimide) (catalog number L001; NanoTemper Technologies, Germany) according to the manufacturer’s instructions. Labeled protein was eluted in 50 mM HEPES, pH 7.5, 100 mM NaCl, 5 mM CaCl_2_, 5 mM TCEP with 0.05% (vol/vol) Tween 20. For analysis of the binding, 16 serial dilutions of each oligosaccharide were prepared in the same buffer (1,000 to 0.03 μM or 100 to 0.003 μM) and mixed 1:1 with labeled BoSGBP_MLG_-A (at a final concentration of 50 or 100 nM). The samples were incubated at room temperature for 15 min and loaded into standard treated capillaries (catalog number MO-KO22; NanoTemper Technologies, Germany). The MST traces were recorded at 25°C (40% light-emitting diode [LED] power and medium MST power) using the MO.Control software version 1.6. Triplicates of independent measurements were analyzed with MO.Affinity analysis software version 2.3 (NanoTemper Technologies, Germany) to calculate the binding affinity expressed as a *K_a_* value.

### ITC.

The thermodynamic parameters of the binding of BoSGBP_MLG_-A and mutants to soluble polysaccharides were quantified by isothermal titration calorimetry (ITC) using a VP-ITC calorimeter (MicroCal, Northampton, MA, USA) at 25°C. All proteins were buffer exchanged to 50 mM HEPES, pH 7.5, 100 mM NaCl, 5 mM CaCl_2_, with 5 mM TCEP, and polysaccharides prepared in the same buffer to minimize heats of dilution. During titration, BoSGBP_MLG_-A proteins (33 to 53 μM) were stirring in the reaction cell (329 rev/min) while being successively injected with 28 pulses (220-s spacing) of 10 μl of polysaccharide (2.5 mg/ml). Integration was corrected by subtracting the value for the carbohydrate titration into the buffer run and analyzed by nonlinear regression using a single-site binding model, and the association constants *K_a_* and binding enthalpy (Δ*H*) were obtained (MicroCal Origin version 7.0; MicroCal Software). The standard Gibbs energy change ΔG° and the standard entropy change ΔS° were calculated using the thermodynamic equation *RT* ln *K_a_* = Δ*G* = Δ*H* − *T*Δ*S*, where *R* is the gas constant and *T* the absolute temperature (K). For polysaccharides, a binding stoichiometry of 1:1 was assumed (*N* = 1) to overcome the problem of converting concentration to molarity.

### DSC.

The structural integrity of recombinant BoSGBP_MLG_-A and mutants was confirmed by differential scanning calorimetry (DSC), using a Nano DSC (TA Instruments, New Castle, DE, USA). Samples were diluted to 0.8 mg/ml in 50 mM HEPES (pH 7.5), 100 mM NaCl, and 5 mM CaCl_2_. DSC scans were performed in a 0.3-ml platinum cell, with temperatures ranging from 10 to 100°C and a scan rate of 0.5°C/min. Sample data were corrected with the buffer’s baseline value, and thermograms generated with NanoAnalyze data analysis software version 3.8.0 (TA Instruments). The measured melting temperatures were not significantly changed (from 53.02 ± 0.01°C for the native BoSGBP_MLG_-A-ZB to a range of 51.75 ± 0.01 to 48.65 ± 0.03°C for mutants), thus reflecting stable folding of the proteins.

### Protein crystallization and X-ray diffraction data collection.

For protein-ligand complex formation, BoSGBP_MLG_-A-ZB (8 mg/ml) was supplemented with 1 mM MgCl_2_ and incubated overnight with barley-9 (G4G3G4G4G3G4G4G3G; 0.33 mM). The BoSGBP_MLG_-A-ZB–barley-9 complex was crystallized by the vapor diffusion method in sitting drops prepared with an automated crystallization nanodrop robot (Oryx8; Douglas Instruments, Hungerford, UK) on 96-well crystallization plates against a reservoir containing 50 μl of precipitant. Drops were set up in a proportion of 2:1, with 0.67 μl of protein-ligand solution mixed with 0.33 μl of reservoir solution, to a maximum volume of 1 μl. Crystals were obtained in 25% polyethylene glycol (PEG) 3350, 0.1 M Bis(2-hydroxyethyl)amino-tris(hydroxymethyl)methane (Bis-Tris), pH 5.5, 0.2 mM MgCl_2_·6H_2_O directly from JCSG-plus screening (Molecular Dimensions, Inc., USA), at room temperature. Crystals of the BO2943-ZB–barley-9 complex were flash-frozen in a cryoprotectant solution containing 28% PEG 3350, 0.1 M Bis-Tris, pH 5.5, 0.2 MgCl_2_·6H_2_O, and 10% (vol/vol) glycerol.

Complete X-ray diffraction data were collected in the I02 beamline at Diamond Light Source (Oxfordshire, UK) to a maximum resolution of 1.4 Å, using radiation of 0.9795-Å wavelength. The crystal of the BoSGBP_MLG_-A-ZB–barley-9 complex was indexed in space group *P*2_1_2_1_2_1_, with unit-cell parameters of *a *= 87.8 Å, *b *= 88.4 Å, *c *= 156.2 Å, and α = β = γ = 90°. Considering the molecular weight of BoSGBP_MLG_-A as 68 kDa, Matthews coefficient (*V*_M_) calculations report a *V*_M_ of 2.24 Å^3^ · Da^−1^, corresponding to 2 monomers of BoSGBP_MLG_-A in the asymmetric unit and a solvent content of 45%. The diffraction data were automatically indexed, integrated, and scaled using the AutoPROC software package (42). Data collection and processing statistics and parameters are summarized in Table 1.

### 3-D structure solution and refinement.

The BoSGBP_MLG_-A–barley-9 crystal structure was solved by molecular replacement with PhaserMR (43), using as the search model the structure of the chitin-binding SusD homolog protein from Flavobacterium johnsoniae (PDB identification code [ID] 5J5U) (44). After preliminary model correction and completion with AutoBuild implemented in Phenix (45), the subsequent refinements were performed using the program phenix.refine (46) from the Phenix platform with alternate cycles of manual model rebuilding with Coot (47). The final refinement was carried out using the web server PDB-REDO (48). The final model, refined to *R*_work_ = 16.38% and *R*_free_ = 18.97%, comprises two polypeptide chains, A and B, of BoSGBP_MLG_-A (residues 40 to 559) in complex with two sugar polymers of 9 β-glucose units and two magnesium ions. The solvent fraction of the final model is constituted by 974 water molecules, 3 sodium ions, one Tris(hydroxyethyl)aminomethane, 14 azide ions, 5 glycerol, and 8 polyethylene glycol molecules. The conformation and stereochemistry of the carbohydrate ligand barley-9 were validated with Privateer (Table S8) (49). Figures were generated with CCP4mg (50), Chimera (51), and ChemDraw (PerkinElmer Informatics). Structure solution and refinement statistics are reported in Table 1.

### Data availability.

Data supporting the findings of the manuscript are available from the corresponding authors upon request. The carbohydrate microarray data are detailed according to MIRAGE guidelines in the supplemental information. Coordinates and structure factors for the BoSGBP_MLG_-A–barley-9 crystal structure have been deposited in the Protein Data Bank under PDB ID 7NOX.

## References

[1] Koropatkin NM, Cameron EA, Martens EC. 2012. How glycan metabolism shapes the human gut microbiota. Nat Rev Microbiol 10:323–335. doi:10.1038/nrmicro2746. 22491358 PMC4005082

[2] El Kaoutari A, Armougom F, Gordon JI, Raoult D, Henrissat B. 2013. The abundance and variety of carbohydrate-active enzymes in the human gut microbiota. Nat Rev Microbiol 11:497–504. doi:10.1038/nrmicro3050. 23748339

[3] Cockburn DW, Koropatkin NM. 2016. Polysaccharide degradation by the intestinal microbiota and its influence on human health and disease. J Mol Biol 428:3230–3252. doi:10.1016/j.jmb.2016.06.021. 27393306

[4] Thaiss CA, Itav S, Rothschild D, Meijer MT, Levy M, Moresi C, Dohnalová L, Braverman S, Rozin S, Malitsky S, Dori-Bachash M, Kuperman Y, Biton I, Gertler A, Harmelin A, Shapiro H, Halpern Z, Aharoni A, Segal E, Elinav E. 2016. Persistent microbiome alterations modulate the rate of post-dieting weight regain. Nature 540:544–551. doi:10.1038/nature20796. 27906159

[5] Schroeder BO, Birchenough GMH, Ståhlman M, Arike L, Johansson MV, Hansson GC, Bäckhed F. 2018. Bifidobacteria or fiber protects against diet-induced microbiota-mediated colonic mucus deterioration. Cell Host Microbe 23:27–40.e7. doi:10.1016/j.chom.2017.11.004. 29276171 PMC5764785

[6] Corfield AP. 2018. The interaction of the gut microbiota with the mucus barrier in health and disease in human. Microorganisms 6:78. doi:10.3390/microorganisms6030078. PMC6163557 30072673

[7] El Khoury D, Cuda C, Luhovyy BL, Anderson GH. 2012. Beta glucan: health benefits in obesity and metabolic syndrome. J Nutr Metab 2012:851362. doi:10.1155/2012/851362. 22187640 PMC3236515

[8] Bashir KM, Choi J-S. 2017. Clinical and physiological perspectives of β-glucans: the past, present, and future. Int J Mol Sci 18:1906. doi:10.3390/ijms18091906. PMC5618555 28872611

[9] Burton RA, Fincher GB. 2009. (1,3;1,4)-β-D-Glucans in cell walls of the Poaceae, lower plants, and fungi: a tale of two linkages. Mol Plant 2:873–882. doi:10.1093/mp/ssp063. 19825664

[10] Pandeirada CO, Maricato É, Ferreira SS, Correia VG, Pinheiro BA, Evtuguin DV, Palma AS, Correia A, Vilanova M, Coimbra MA, Nunes C. 2019. Structural analysis and potential immunostimulatory activity of Nannochloropsis oculata polysaccharides. Carbohydr Polym 222:114962. doi:10.1016/j.carbpol.2019.06.001. 31320077

[11] Lazaridou A, Biliaderis CG, Micha-Screttas M, Steele BR. 2004. A comparative study on structure-function relations of mixed-linkage (1→3), (1→4) linear β-D-glucans. Food Hydrocoll 18:837–855. doi:10.1016/j.foodhyd.2004.01.002.

[12] Kiemle SN, Zhang X, Esker AR, Toriz G, Gatenholm P, Cosgrove DJ. 2014. Role of (1,3)(1,4)-β-glucan in cell walls: interaction with cellulose. Biomacromolecules 15:1727–1736. doi:10.1021/bm5001247. 24678830

[13] Martens EC, Lowe EC, Chiang H, Pudlo NA, Wu M, McNulty NP, Abbott DW, Henrissat B, Gilbert HJ, Bolam DN, Gordon JI. 2011. Recognition and degradation of plant cell wall polysaccharides by two human gut symbionts. PLoS Biol 9:e1001221. doi:10.1371/journal.pbio.1001221. 22205877 PMC3243724

[14] Tamura K, Hemsworth GR, Déjean G, Rogers TE, Pudlo NA, Urs K, Jain N, Davies GJ, Martens EC, Brumer H. 2017. Molecular mechanism by which prominent human gut bacteroidetes utilize mixed-linkage beta-glucans, major health-promoting cereal polysaccharides. Cell Rep 21:417–430. doi:10.1016/j.celrep.2017.09.049. 29020628 PMC5656003

[15] Tamura K, Foley MH, Gardill BR, Dejean G, Schnizlein M, Bahr CME, Louise Creagh A, van Petegem F, Koropatkin NM, Brumer H. 2019. Surface glycan-binding proteins are essential for cereal beta-glucan utilization by the human gut symbiont Bacteroides ovatus. Cell Mol Life Sci 76:4319–4340. doi:10.1007/s00018-019-03115-3. 31062073 PMC6810844

[16] Rillahan CD, Paulson JC. 2011. Glycan microarrays for decoding the glycome. Annu Rev Biochem 80:797–823. doi:10.1146/annurev-biochem-061809-152236. 21469953 PMC3116967

[17] Palma AS, Feizi T, Childs RA, Chai W, Liu Y. 2014. The neoglycolipid (NGL)-based oligosaccharide microarray system poised to decipher the meta-glycome. Curr Opin Chem Biol 18:87–94. doi:10.1016/j.cbpa.2014.01.007. 24508828 PMC4105633

[18] Geissner A, Seeberger PH. 2016. Glycan arrays: from basic biochemical research to bioanalytical and biomedical applications. Annu Rev Anal Chem (Palo Alto Calif) 9:223–247. doi:10.1146/annurev-anchem-071015-041641. 27306309

[19] Ribeiro DO, Pinheiro BA, Carvalho AL, Palma AS. 2018. Targeting protein-carbohydrate interactions in plant cell-wall biodegradation: the power of carbohydrate microarrays, p 159–176. In Rauter AP, Lindhorst T, Queneau Y (ed), Carbohydrate chemistry: chemical and biological approaches, 1st ed, vol 43. Royal Society of Chemistry, London, UK.

[20] Palma AS, Liu Y, Zhang H, Zhang Y, McCleary BV, Yu G, Huang Q, Guidolin LS, Ciocchini AE, Torosantucci A, Wang D, Carvalho AL, Fontes CMGA, Mulloy B, Childs RA, Feizi T, Chai W. 2015. Unravelling glucan recognition systems by glycome microarrays using the designer approach and mass spectrometry. Mol Cell Proteomics 14:974–988. doi:10.1074/mcp.M115.048272. 25670804 PMC4390274

[21] Zhang H, Palma AS, Zhang Y, Childs RA, Liu Y, Mitchell DA, Guidolin LS, Weigel W, Mulloy B, Ciocchini AE, Feizi T, Chai W. 2016. Generation and characterization of β1,2-gluco-oligosaccharide probes from Brucella abortus cyclic β-glucan and their recognition by C-type lectins of the immune system. Glycobiology 26:1086–1096. doi:10.1093/glycob/cww041. 27053576 PMC5072146

[22] Ribeiro DO, Viegas A, Pires VMR, Medeiros‐Silva J, Bule P, Chai W, Marcelo F, Fontes CMGA, Cabrita EJ, Palma AS, Carvalho AL. 2020. Molecular basis for the preferential recognition of β1,3‐1,4‐glucans by the family 11 carbohydrate‐binding module from Clostridium thermocellum. FEBS J 287:2723–2743. doi:10.1111/febs.15162. 31794092

[23] Kadirvelraj R, Foley BL, Dyekjaer JD, Woods RJ. 2008. Involvement of water in carbohydrate−protein binding: concanavalin A revisited. J Am Chem Soc 130:16933–16942. doi:10.1021/ja8039663. 19053475 PMC2626182

[24] Schiebel J, Gaspari R, Wulsdorf T, Ngo K, Sohn C, Schrader TE, Cavalli A, Ostermann A, Heine A, Klebe G. 2018. Intriguing role of water in protein-ligand binding studied by neutron crystallography on trypsin complexes. Nat Commun 9:3559. doi:10.1038/s41467-018-05769-2. 30177695 PMC6120877

[25] Darby JF, Hopkins AP, Shimizu S, Roberts SM, Brannigan JA, Turkenburg JP, Thomas GH, Hubbard RE, Fischer M. 2019. Water networks can determine the affinity of ligand binding to proteins. J Am Chem Soc 141:15818–15826. doi:10.1021/jacs.9b06275. 31518131

[26] Vergara R, Romero‐Romero S, Velázquez‐López I, Espinoza‐Pérez G, Rodríguez‐Hernández A, Pulido NO, Sosa‐Peinado A, Rodríguez‐Romero A, Fernández‐Velasco DA. 2020. The interplay of protein–ligand and water‐mediated interactions shape affinity and selectivity in the LAO binding protein. FEBS J 287:763–782. doi:10.1111/febs.15019. 31348608

[27] Martens EC, Koropatkin NM, Smith TJ, Gordon JI. 2009. Complex glycan catabolism by the human gut microbiota: the Bacteroidetes sus-like paradigm. J Biol Chem 284:24673–24677. doi:10.1074/jbc.R109.022848. 19553672 PMC2757170

[28] Glenwright AJ, Pothula KR, Bhamidimarri SP, Chorev DS, Baslé A, Firbank SJ, Zheng H, Robinson CV, Winterhalter M, Kleinekathöfer U, Bolam DN, Van Den Berg B. 2017. Structural basis for nutrient acquisition by dominant members of the human gut microbiota. Nature 541:407–411. doi:10.1038/nature20828. 28077872 PMC5497811

[29] Gray DA, White JBR, Oluwole AO, Rath P, Glenwright AJ, Mazur A, Zahn M, Baslé A, Morland C, Evans SL, Cartmell A, Robinson CV, Hiller S, Ranson NA, Bolam DN, van den Berg B. 2021. Insights into SusCD-mediated glycan import by a prominent gut symbiont. Nat Commun 12:44. doi:10.1038/s41467-020-20285-y. 33398001 PMC7782687

[30] Tauzin AS, Kwiatkowski KJ, Orlovsky NI, Smith CJ, Creagh AL, Haynes CA, Wawrzak Z, Brumer H, Koropatkin NM. 2016. Molecular dissection of xyloglucan recognition in a prominent human gut symbiont. mBio 7:e02134-15. doi:10.1128/mBio.02134-15. 27118585 PMC4850273

[31] Rogowski A, Briggs JA, Mortimer JC, Tryfona T, Terrapon N, Lowe EC, Baslé A, Morland C, Day AM, Zheng H, Rogers TE, Thompson P, Hawkins AR, Yadav MP, Henrissat B, Martens EC, Dupree P, Gilbert HJ, Bolam DN. 2015. Glycan complexity dictates microbial resource allocation in the large intestine. Nat Commun 6:7481. doi:10.1038/ncomms8481. 26112186 PMC4491172

[32] Cuskin F, Lowe EC, Temple MJ, Zhu Y, Cameron E, Pudlo NA, Porter NT, Urs K, Thompson AJ, Cartmell A, Rogowski A, Hamilton BS, Chen R, Tolbert TJ, Piens K, Bracke D, Vervecken W, Hakki Z, Speciale G, Munōz-Munōz JL, Day A, Peña MJ, McLean R, Suits MD, Boraston AB, Atherly T, Ziemer CJ, Williams SJ, Davies GJ, Abbott DW, Martens EC, Gilbert HJ. 2015. Human gut Bacteroidetes can utilize yeast mannan through a selfish mechanism. Nature 517:165–169. doi:10.1038/nature13995. 25567280 PMC4978465

[33] Rahman O, Cummings SP, Harrington DJ, Sutcliffe IC. 2008. Methods for the bioinformatic identification of bacterial lipoproteins encoded in the genomes of Gram-positive bacteria. World J Microbiol Biotechnol 24:2377–2382. doi:10.1007/s11274-008-9795-2.

[34] Petersen TN, Brunak S, von Heijne G, Nielsen H. 2011. SignalP 4.0: discriminating signal peptides from transmembrane regions. Nat Methods 8:785–786. doi:10.1038/nmeth.1701. 21959131

[35] Li MZ, Elledge SJ. 2007. Harnessing homologous recombination in vitro to generate recombinant DNA via SLIC. Nat Methods 4:251–256. doi:10.1038/nmeth1010. 17293868

[36] Sequeira AF, Brás JLA, Fernandes VO, Guerreiro CIPD, Vincentelli R, Fontes CMGA. 2017. A novel platform for high-throughput gene synthesis to maximize recombinant expression in Escherichia coli. 1620:113–128. Methods Mol Biol. doi:10.1007/978-1-4939-7060-5_7. 28540703

[37] Palma AS, Liu Y, Muhle-Goll C, Butters TD, Zhang Y, Childs R, Chai W, Feizi T. 2010. Multifaceted approaches including neoglycolipid oligosaccharide microarrays to ligand discovery for malectin. Methods Enzymol 478:265–286. doi:10.1016/S0076-6879(10)78013-7. 20816485

[38] Liu Y, McBride R, Stoll M, Palma AS, Silva L, Agravat S, Aoki-Kinoshita KF, Campbell MP, Costello CE, Dell A, Haslam SM, Karlsson NG, Khoo K-H, Kolarich D, Novotny MV, Packer NH, Ranzinger R, Rapp E, Rudd PM, Struwe WB, Tiemeyer M, Wells L, York WS, Zaia J, Kettner C, Paulson JC, Feizi T, Smith DF. 2017. The minimum information required for a glycomics experiment (MIRAGE) project: improving the standards for reporting glycan microarray-based data. Glycobiology 27:280–284. doi:10.1093/glycob/cww118. 27993942 PMC5444268

[39] Liu Y, Palma AS, Feizi T, Chai W. 2018. Insights into glucan polysaccharide recognition using glucooligosaccharide microarrays with oxime-linked neoglycolipid probes. Methods Enzymol 598:139–167. doi:10.1016/bs.mie.2017.09.001. 29306433

[40] Liu Y, Childs RA, Palma AS, Campanero-Rhodes MA, Stoll MS, Chai W, Feizi T. 2012. Neoglycolipid-based oligosaccharide microarray system: preparation of NGLs and their noncovalent immobilization on nitrocellulose-coated glass slides for microarray analyses. Methods Mol Biol 808:117–136. doi:10.1007/978-1-61779-373-8_8. 22057521

[41] Stoll M, Feizi T. 2009. Software tools for storing, processing and displaying carbohydrate microarray data, p 123–140. *In* Hicks MG, Kettner C (ed), Glyco-bioinformatics: bits ‘n’ bytes of sugars. Proceeding of the International Beilstein symposium. Beilstein Institute for the Advancement of Chemical Sciences, Frankfurt, Germany.

[42] Vonrhein C, Flensburg C, Keller P, Sharff A, Smart O, Paciorek W, Womack T, Bricogne G. 2011. Data processing and analysis with the autoPROC toolbox. Acta Crystallogr D Biol Crystallogr 67:293–302. doi:10.1107/S0907444911007773. 21460447 PMC3069744

[43] McCoy AJ, Grosse-Kunstleve RW, Adams PD, Winn MD, Storoni LC, Read RJ. 2007. Phaser crystallographic software. J Appl Crystallogr 40:658–674. doi:10.1107/S0021889807021206. 19461840 PMC2483472

[44] Larsbrink J, Zhu Y, Kharade SS, Kwiatkowski KJ, Eijsink VGHH, Koropatkin NM, McBride MJ, Pope PB. 2016. A polysaccharide utilization locus from Flavobacterium johnsoniae enables conversion of recalcitrant chitin. Biotechnol Biofuels 9:260. doi:10.1186/s13068-016-0674-z. 27933102 PMC5127042

[45] Liebschner D, Afonine PV, Baker ML, Bunkoczi G, Chen VB, Croll TI, Hintze B, Hung LW, Jain S, McCoy AJ, Moriarty NW, Oeffner RD, Poon BK, Prisant MG, Read RJ, Richardson JS, Richardson DC, Sammito MD, Sobolev OV, Stockwell DH, Terwilliger TC, Urzhumtsev AG, Videau LL, Williams CJ, Adams PD. 2019. Macromolecular structure determination using X-rays, neutrons and electrons: recent developments in Phenix. Acta Crystallogr D Struct Biol 75:861–877. doi:10.1107/S2059798319011471. 31588918 PMC6778852

[46] Afonine PV, Grosse-Kunstleve RW, Echols N, Headd JJ, Moriarty NW, Mustyakimov M, Terwilliger TC, Urzhumtsev A, Zwart PH, Adams PD. 2012. Towards automated crystallographic structure refinement with phenix.refine. Acta Crystallogr D Biol Crystallogr 68:352–367. doi:10.1107/S0907444912001308. 22505256 PMC3322595

[47] Emsley P, Lohkamp B, Scott WG, Cowtan K. 2010. Features and development of Coot. Acta Crystallogr D Biol Crystallogr 66:486–501. doi:10.1107/S0907444910007493. 20383002 PMC2852313

[48] Joosten RP, Long F, Murshudov GN, Perrakis A. 2014. The PDB_REDO server for macromolecular structure model optimization. IUCrJ 1(Pt 4):213–220. doi:10.1107/S2052252514009324. PMC4107921 25075342

[49] Agirre J, Iglesias-Fernández J, Rovira C, Davies GJ, Wilson KS, Cowtan KD. 2015. Privateer: software for the conformational validation of carbohydrate structures. Nat Struct Mol Biol 22:833–834. doi:10.1038/nsmb.3115. 26581513

[50] McNicholas S, Potterton E, Wilson KS, Noble MEM. 2011. Presenting your structures: the CCP 4 mg molecular-graphics software. Acta Crystallogr D Biol Crystallogr 67:386–394. doi:10.1107/S0907444911007281. 21460457 PMC3069754

[51] Pettersen EF, Goddard TD, Huang CC, Couch GS, Greenblatt DM, Meng EC, Ferrin TE. 2004. UCSF Chimera—a visualization system for exploratory research and analysis. J Comput Chem 25:1605–1612. doi:10.1002/jcc.20084. 15264254

[52] Neelamegham S, Aoki-Kinoshita K, Bolton E, Frank M, Lisacek F, Lütteke T, O’Boyle N, Packer NH, Stanley P, Toukach P, Varki A, Woods RJ, Darvill A, Dell A, Henrissat B, Bertozzi C, Hart G, Narimatsu H, Freeze H, Yamada I, Paulson J, Prestegard J, Marth J, Vliegenthart JFG, Etzler M, Aebi M, Kanehisa M, Taniguchi N, Edwards N, Rudd P, Seeberger P, Mazumder R, Ranzinger R, Cummings R, Schnaar R, Perez S, Kornfeld S, Kinoshita T, York W, Knirel Y, SNFG Discussion Group. 2019. Updates to the Symbol Nomenclature for Glycans guidelines. Glycobiology 29:620–624. doi:10.1093/glycob/cwz045. 31184695 PMC7335484

