# Supplementary material for: Mapping Molecular Recognition of β1,3-1,4-Glucans by a Surface Glycan-Binding Protein from the Human Gut Symbiont Bacteroides ovatus
Source: Microbiol Spectr. 2021 Nov 24;9(3):e01826-21. doi: 10.1128/Spectrum.01826-21 (PMC8612152; doi:10.1128/Spectrum.01826-21)
Supplement: SUPPLEMENTAL FILE 1 — Supplemental material. Download SPECTRUM01826-21_Supp_1_seq10.pdf, PDF file, 0.8 MB [file spectrum01826-21_supp_1_seq10.pdf]

Supplemental Material

Mapping molecular recognition of β1,3-1,4-glucans by a surface glycan-binding protein from the human gut symbiont *Bacteroides ovatus*

Viviana G. Correia[a,b], Filipa Trovão[a,b], Benedita A. Pinheiro[a,b], Joana L. A. Brás[c], Lisete M. Silva[d]*, Cláudia Nunes[e], Manuel A. Coimbra[f], Yan Liu[d], Ten Feizi[d], Carlos M. G. A. Fontes[c,g], Barbara Mulloy[d], Wengang Chai[d], Ana Luísa Carvalho[a,b#], Angelina S. Palma[a,b#]

[a] UCIBIO, Applied Molecular Biosciences Unit, Department of Chemistry, School of Science and Technology, NOVA University Lisbon, Caparica, Portugal

[b] Associate Laboratory i4HB - Institute for Health and Bioeconomy, School of Science and Technology, NOVA University Lisbon, Caparica, Portugal

[c] NZYTech Genes & Enzymes, Lisbon, Portugal

[d] Glycosciences Laboratory, Department of Metabolism Digestion and Reproduction, Imperial College London, London, United Kingdom

[e] CICECO, Department of Chemistry, University of Aveiro, Aveiro, Portugal

[f] LAQV-REQUIMTE, Department of Chemistry, University of Aveiro, Aveiro, Portugal

[g] CIISA, Faculty of Veterinary Medicine, University of Lisbon, Lisbon, Portugal

# Address correspondence to Angelina S. Palma, angelina.palma@fct.unl.pt, and Ana Luísa Carvalho, almc@fct.unl.pt.

* Present address: LAQV-REQUIMTE, Department of Chemistry, University of Aveiro, Aveiro, Portugal

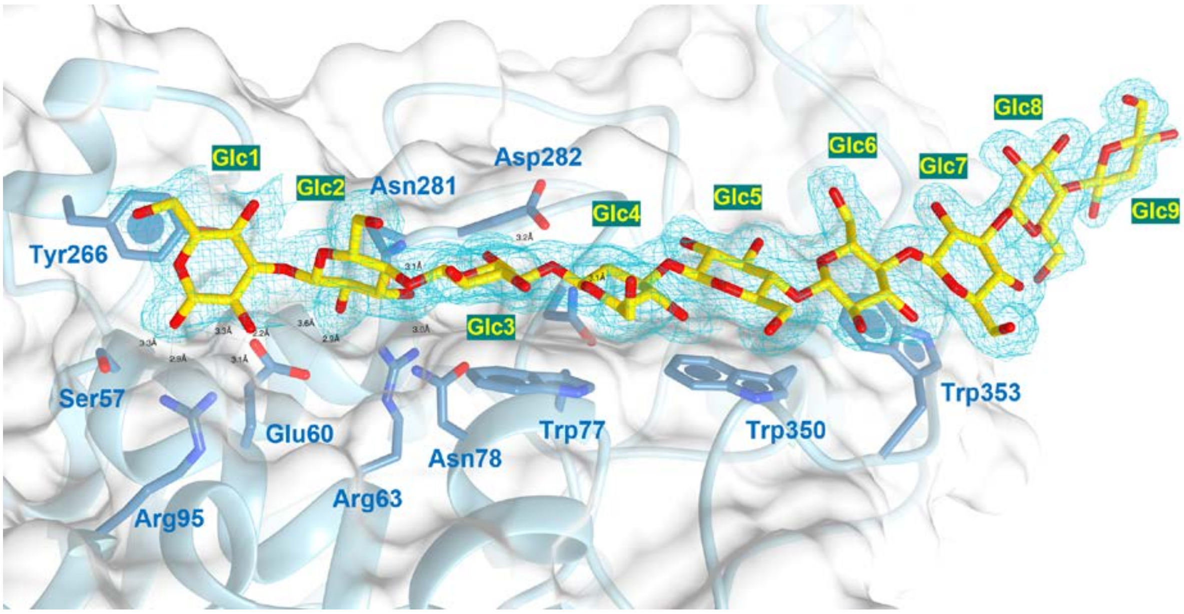

Supplemental Figure S1. Close-up view of the binding site of $BoSGBP_{MLG}$-A (in light blue ribbon and white transparent surface) accommodating the barley-9 (G4G3G4G4G3G4G4G3G, in stick representation with yellow carbon atoms and red oxygen atoms). The side-chains of the protein residues involved in binding are shown as stick model and labelled in blue. Carbon atoms are in blue, nitrogen atoms in dark blue and oxygen atoms in red. The final $2mF_{obs}$-DF_{calc} electron density map is shown around the ligand and contoured at 0.9 rmsd. Amino acids are numbered according to the native protein. Glucose units (Glc) are numbered from the reducing to the non-reducing end.

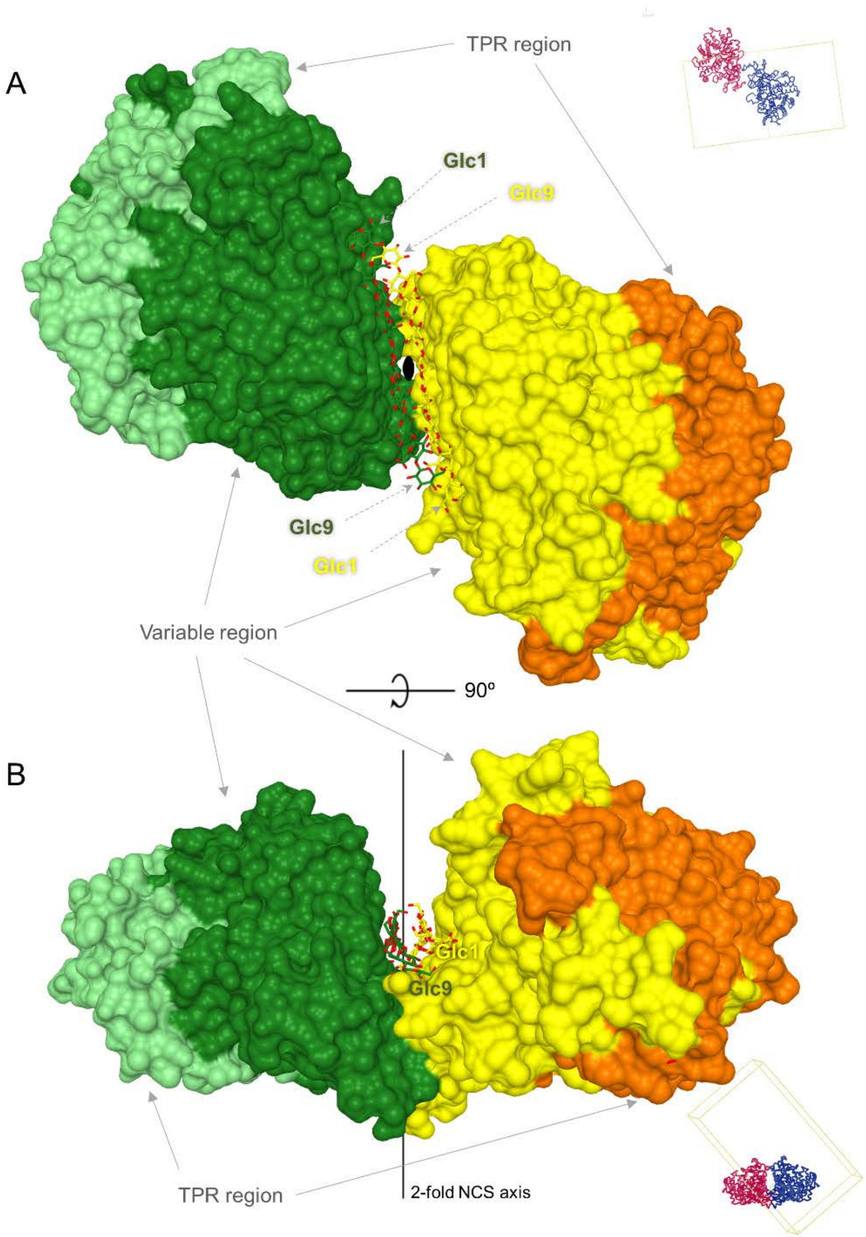

Supplemental Figure S2. Protein-sugar-sugar-protein supramolecular assembly observed in the asymmetric unit of the BoSGBP$_{MLG}$-A-barley-9 complex 3D structure. Top (A) and side (B) view along the two-fold NCS axis between the two BoSGBP$_{MLG}$-A-barley-9 complexes in the asymmetric unit. The variable regions (dark green and yellow surfaces) of the protein monomers are binding the outer faces of two dimerized barley-9 antiparallel chains, forming a protein-sugar-sugar-protein supramolecular assembly. The TPR region of each protein monomer is shown in light green and orange surfaces. Each barley-9 chain is represented as stick model and the carbon atoms are coloured according to the corresponding protein monomer surface. Glucose residues 1 (Glc1, reducing end) and 9 (Glc9, non-reducing end) of each barley-9 chain are labelled and indicated by arrows.

Supplemental Table S1. Saccharide probes (polysaccharides and glycoproteins) included in the fungal, bacterial, microalgae and plant saccharide microarray, sorted by backbone type and main glycosidic linkage.

ID[a]	Saccharide Probe[b]	Biological Source & Supplier[c]	Predominant oligosaccharide sequence/Monosaccharide composition	Reference[d]
1	Dextran	*Leuconostoc mesenteroides*; Sigma-Aldrich (D4876)	α1,6-Glc	(1, 2)
2	Pullulan	*Aureobasidium pullulans*; Megazyme (P-PULLN)	Mixed-linked α1,4-1,6-Glc (α1,6-linked maltotriosyl repeats)	(2, 3)
3	Curdlan*	*Agrobacterium* sp., strain ATCC31749	β1,3-Glc	(2, 4)
4	NSG-β-glucan (Neutral soluble glucan)	*Saccharomyces cerevisiae;* Biothera	β1,3-Glc backbone with occasional monoglucosyl β1,6-Glc ramifications	(2, 5)
5	PGG-β-glucan (Poly-(1,6)-D-glucopyranosyl-(1,3)-D-glucopyranose)	*Saccharomyces cerevisiae;* Biothera	β1,3-Glc backbone with occasional monoglucosyl β1,6-Glc ramifications	(2, 6)
6	Lentinan	*Lentinus edodes*	β1,3-Glc backbone with occasional monoglucosyl β1,6-Glc ramifications	(2, 7)
7	Grifolan	*Grifola frondosa*	β1,3-Glc backbone with highly ramified oligomeric β1,6-Glc ramifications	(2, 8)
8	Pustulan	*Umbilicaria papullosa*; Elicityl (GLU900)	β1,6-Glc	(2, 9)
9	β-glucan (Barley)	Barley flour; Megazyme (P-BGBL)	Mixed-linked β1,3-1,4-Glc; 1:3-4 linkage ratio; contains Ara (2%), Xyl (0.2%)	(2, 10)
10	Lichenan	Icelandic moss; Megazyme (P-LICHN)	Mixed-linked β1,3-1,4-Glc; 1:2 linkage ratio	(11)
11	Microalgae fraction (*N.oculata*)	*Nannochloropsis oculata* Et50 enriched polysaccharide fraction	Mixed-linked β1,3-1,4-Glc; Rha (5%), Fuc (1%), Rib (35%), Ara (2%), Xyl (2%), Man (5%), Gal (1%), Glc (34%), Uronic Acids (UA, 11%)	(12)
12	Xyloglucan (Plum fresh)	Fresh plum *Prunus domestica L.*	β1,4-Glc backbone with α1,6-Xyl ramifications, substituted with β1,2-Gal or β1,2-Gal capped with α1,2-Fuc; Rha (2%), Fuc (5%), Ara (5%), Xyl (40%), Man (6%), Gal (13%), Glc(24%), UA (6%)	(13)
13	Xyloglucan (Plum boiled)	Boiled plum *Prunus domestica L.*	β1,4-Glc backbone with α1,6-Xyl ramifications, substituted with β1,2-Gal or β1,2-Gal capped with α1,2-Fuc; Rha (2%), Fuc (6%), Ara (6%), Xyl (46%), Man (4%), Gal (14%), Glc (22%), UA (1%)	(13)
14	Xylan (*P. palmata*)	*Palmaria palmata*; Elicityl (XYL100)	Mixed-linked β1,3-β1,4-D-Xyl; 1:4 linkage ratio	
15	Xylan (Plum)	Plum *Prunus domestica L.*	Mixed-linked β1,3-β1,4-D-Xyl; Rha (8%), Ara (13%), Xyl (69%), Man (2%), Gal (3%), Glc (1%), UA (3%)	(13)
16	Arabinoxylan	Dreche (brewers' spent grain)	β1,4-Xyl backbone with α1,4-Ara ramifications; Ara (40%), Xyl (54%), Man (0%), Gal (3%), Glc (3%)	(14)
17	Galactomannan (Guar)	Guar; Megazyme (P-GGMMV)	β1,4-Man backbone with α1,6-Gal ramifications; Gal (38%), Man (62%)	
18	Galactomannan (Guar, ΔGal)	Guar; Gal depleted; Megazyme (P-GGM21)	β1,4-Man backbone with α1,6-Gal ramifications; Gal (21%), Man (79%)	
19	Galactomannan (Carob)	Carob; Megazyme (P-GALML)	β1,4-Man backbone with α1,6-Gal ramifications; Gal (24%), Man (76%)	
20	Galacturonate (Apple)	Apple pectins; Low methylated; Elicityl (GAT100)	α1,4-GalA backbone with α1,2-Rha and other side chains	

21	Galacturonate (Citrus)	Citrus pectins; Low methylated; Elicityl (GAT102)	α1,4-GalA backbone with α1,2-Rha and other side chains; Ara (0.21%), Rha (0.42%), Fuc (0.01%), Xyl (1.5%), Gal (3.67%), Glc (0.94%), UA (92.6%)	
22	Polygalacturonic Acid (PGA) (Citrus)	Citrus; Megazyme (P-PGACT)	α1,4-GalA backbone with possible ramifications; Ara (0.81%), Rha (0.47%), Fuc (0.01%), Xyl (0.09%), Gal (4.51%), Glc (3.03%), UA (88.6%)	
23	Rhamnogalacturonan (Soybean)	Soybean; Megazyme (P-RHAGN)	Mixed-linked α1,4-GalA and α1,2-Rha backbone with ramifications; GalA (51%), Rha(13%), Fuc (21%), Ara (7%), Xyl (28%), Gal (25%), other neutral sugars (3%)	
24	Galactan (Lupin)	Lupin; Arabinofuranosidase treated pectic galactan; Megazyme (P-GALLU)	β1,4-Gal backbone with possible ramifications; Neutral sugars: Gal (82%), Ara (5.8%), Rha (5.1%), Xyl (1.4%), other (5.7%); and GalA (14.6%)	
25	Pectic Galactan (Lupin)	Lupin; Megazyme (P-PGALU)	β1,4-Gal backbone with possible ramifications; Gal (77%), Ara (14%), Rha (3%), Xyl (0.6%), GalA (5.4%)	
26	Pectic Galactan (Potato)	Potato; Megazyme (P-PGAPT)	β1,4-Gal backbone with possible ramifications; Gal (78%), Ara (9%), Rha (4%), GalA (9%)	
27	Mannan (*S. cerevisiae*)	*Saccharomyces cerevisiae;* Sigma-Aldrich (M7504)	α1,6-Man backbone with oligomeric α1,2-1,3-Man ramifications	(2, 15)
28	Mannan (*C. albicans*)	*Candida albicans*	α1,6-Man backbone with oligomeric α1,2-, α1,3-, and β-1,2-Man ramifications	(11, 15)
29	Lipomannan (LM) (*M. tuberculosis*)	*Mycobacterium tuberculosis*, strain H37Rv; BEI Resources (NR-14850)	Linear α1,6-Man backbone with single mannosyl α1,2-Man ramifications	(16, 17)
30	Lipoarabinomannan (LAM) (*M. tuberculosis*)	*Mycobacterium tuberculosis*, strain H37Rv; BEI Resources (NR-14848)	Linear α1,6-Man backbone with single mannosyl α1,2-Man ramifications and α1,5-Ara polymer ramifications at certain positions with α1,3-1,5-Ara residues, that in turn are terminated by β1,2-Ara and capped by α1,2-Man units	(16, 17)
31	Native O-glycoprotein (*M. tuberculosis*)	*Mycobacterium tuberculosis*, strain H37Rv; BEI Resources (NR-14862)	α1,2-Man	(16, 18)
32	Glucurono-XyloMannan*	*Tremella fuciformi*; Elicityl (HGL200)	α1,3-Man backbone with Xyl, GlcA and Fuc ramifications	

[a] ID number corresponding to the probe position in the binding chart or heatmap.
[b] Probes are grouped according to predominant oligosaccharide sequence and glycosidic linkage.
[c] Sources are indicated for each carbohydrate sample; if commercial, the product code is in brackets.
[d] Reference for the structural analysis or recent published work for each sample, if available.
* Curdlan polysaccharide was solubilized in 50mM NaOH and Glucurono-XyloMannan in 150 mM NaCl, prior printing.

Supplemental Table S2. Fluorescence binding intensities elicited with BoSGBP$_{MLG}$-A, CBMs, antibodies and lectins in the fungal, bacterial, microalgae and plant saccharide microarray.

ID[a]	Probe[b]	BoSGBP$_{MLG}$-A Score ±Err[c]	*h*Malectin Score ±Err[c]	*Tm*CBM41 Score ±Err[c]	BS400-2 β1,3-Glucan Score ±Err[c]	*m*Dectin-1 Score ±Err[c]	BS400-3 β1,3/1,4-Glucan Score ±Err[c]	*Ct*CBM11 Score ±Err[c]	LM25 Xyloglucan Score ±Err[c]	LM11 Xylan/Arabinoxylan Score ±Err[c]	BS400-4 β1,4-Mannan Score ±Err[c]	LM21 Heteromannan Score ±Err[c]	LM6 α1,5-Arabinan Score ±Err[c]	LM5 β1,4-Galactan Score ±Err[c]	ConA Score ±Err[c]	AAL Score ±Err[c]
1	Dextran	-	34148 ±1417	-	-	-	-	-	-	-	-	-	-	-	-	-
2	Pullulan	-	-	63330 ±77	-	-	-	-	-	-	-	-	-	-	-	-
3	Curdlan	-	-	-	25621 ±670	10118 ±121	-	-	-	-	-	-	-	-	-	-
4	NSG-β-glucan (*S.cerevisiae*)	-	-	-	4311 ±108	6147 ±37	-	-	-	-	-	-	-	-	-	-
5	PGG-β-glucan (*S.cerevisiae*)	-	-	3690 ±68	11679 ±824	33693 ±768	-	-	-	-	-	-	-	-	-	-
6	Lentinan	-	-	2047 ±140	1402 ±26	28055 ±13	-	-	-	-	-	-	-	-	-	7528 ±224
7	Grifolan	-	-	-	-	1167 ±79	-	-	-	-	-	-	-	-	-	3056 ±33
8	Pustulan	-	-	-	-	-	-	-	-	-	-	-	-	-	5178 ±283	-
9	β-glucan (Barley)	31878 ±635	-	-	-	-	21768 ±958	13334 ±12	-	-	-	-	-	-	-	-
10	Lichenan	32919 ±5	-	33257 ±294	-	-	21612 ±551	3962 ±154	-	-	-	-	-	-	-	-
11	Microalgae fraction (*N.oculata*)	25046 ±124	-	-	-	-	50231 ±244	55380 ±2927	-	-	-	-	-	-	2807 ±123	4652 ±34
12	Xyloglucan (Plum fresh)	14038 ±395	-	-	1095 ±58	-	-	-	45910 ±1018	-	15549 ±1605	6993 ±137	-	-	-	58791 ±43
13	Xyloglucan (Plum boiled)	16519 ±103	-	-	2252 ±38	-	-	-	49112 ±501	1249 ±28	5318 ±45	8087 ±268	2548 ±8	4031 ±7	-	58119 ±41
14	Xylan (*P.palmata*)	-	-	-	-	-	-	-	-	25072 ±1070	-	-	-	-	-	-
15	Xylan (Plum)	-	-	-	-	-	-	-	2431 ±29	16278 ±592	-	2866 ±42	-	4104 ±132	-	2572 ±54

16	Arabinoxylan	2323 ±169	-	-	-	-	7234 ±47	1045 ±39	8780 ±84	18874 ±286	-	-	13325 ±410	2815 ±115	1448 ±40	26868 ±1189
17	Galactomannan (Guar)	-	-	-	-	-	-	-	-	-	51305 ±241	33893 ±300	-	-	-	-
18	Galactomannan (Guar, ΔGal)	-	-	-	-	-	-	-	-	-	50305 ±109	29974 ±83	-	-	-	-
19	Galactomannan (Carob)	-	-	-	-	-	-	-	-	-	50128 ±165	35300 ±1421	1435 ±65	-	-	-
20	Galacturonate (Apple)	-	-	-	-	-	-	-	16518 ±150	-	-	-	-	23365 ±583	-	1214 ±83
21	Galacturonate (Citrus)	-	-	-	-	-	-	-	2013 ±25	-	-	-	-	21305 ±196	-	-
22	Polygalacturonic Acid (Citrus)	-	-	-	-	-	-	-	4151 ±60	-	-	-	4022 ±107	26822 ±649	-	-
23	Rhamnogalacturona n (Soybean)	-	-	-	-	-	-	-	11441 ±588	-	-	1755 ±213	24137 ±305	32192 ±389	1593 ±80	42836 ±289
24	Galactan (Lupin)	-	-	-	-	-	-	-	15305 ±9	-	-	-	32418 ±733	39469 ±1865	-	14618 ±91
25	Pectic Galactan (Lupin)	-	-	-	-	-	-	-	13354 ±132	-	-	-	32066 ±1503	30088 ±1579	-	13749 ±524
26	Pectic Galactan (Potato)	-	-	-	-	-	-	-	-	-	-	-	28122 ±299	36148 ±454	-	2079 ±63
27	Mannan (*S.cerevisiae*)	-	-	-	-	-	-	-	-	-	-	-	-	-	51491 ±1352	-
28	Mannan (*C.albicans*)	-	-	-	1326 ±33	-	-	-	-	-	-	-	-	-	59480 ±264	-
29	Lipomannan (*M.tuberculosis*)	-	-	-	-	-	-	-	-	-	-	-	-	-	59906 ±152	-
30	Lipoarabinomannan (*M.tuberculosis*)	-	-	2185 ±127	-	-	-	-	-	-	-	-	-	-	60424 ±20	-
31	Native O-glycoprotein (*M.tuberculosis*)	-	-	13142 ±197	-	-	-	-	-	-	-	-	-	-	60512 ±43	-
32	Glucurono-XyloMannan	-	-	-	-	-	-	-	-	-	-	-	-	-	1349 ±133	30144 ±2397

[a] ID number corresponding to the probe position in the heatmap.
[b] Carbohydrate probes are all polysaccharides, organized by predominant oligosaccharide composition and glycosidic linkage.
[c] Fluorescence binding signals are shown as means of duplicate spots at 150pg/spot of each carbohydrate probe with respective error (Err).
(-) Binding signal below background (<1000).

Supplemental Table S3. Reported carbohydrate binding and sources of monoclonal antibodies, bacterial CBMs and lectins used as validation and control proteins.

Proteins	Source	Reported carbohydrate binding	Reference
**Monoclonal Antibodies**			
BS400-2 β1,3-Glucan	Mouse IgG; Biosupplies (400-2)	Linear β1,3-Glc oligosaccharide segments in β1,3-D-glucans	(19)
BS400-3 β1,3/1,4-Glucan	Mouse IgG; Biosupplies (400-3)	Linear β1,3-1,4-Glc oligosaccharide segments in mixed-linked β1,3-1,4-D-glucans	(20)
BS400-4 β1,4-Mannan	Mouse IgG; Biosupplies (400-4)	Linear β1,4-Man oligosaccharides segments in β1,4-D-mannans and -galactomannans	(21)
LM5 β1,4-Galactan	Rat IgG; Plant probes (LM5-050)	β1-4-galactosyl residues found in the galactan components of certain pectic polymers; Linear tetrasaccharide in β1,4-D-galactan	(22)
LM6 α1,5-Arabinan	Rat IgG; Plant probes (LM6-050)	α1,5-L-arabinan polysaccharides and α1,5-Ara oligosaccharides; α1,5-Ara residues found in the arabinan components of certain pectic polymers; Arabinogalactan-proteins	(23)
LM11 Xylan/Arabinoxylan	Rat IgM; Plant probes (LM11)	β1,4-Xyl backbone unsubstituted or substituted with α-arabinose sidechains in xylans and arabinoxylans	(24)
LM21 Heteromannan	Rat IgM; Plant probes (LM21)	β1,4-Man in mannan, glucomannan and galactomannan polysaccharides; β1,4 manno-oligosaccharides (DP2-DP5)	(25)
LM25 Xyloglucan	Rat IgM; Plant probes (LM25)	Xyloglucan polysaccharides; XLLG, XXLG and XXXG oligosaccharides	(26)
**Bacterial CBMs**			
*Cm*CBM6-2	*Cellvibrio mixtus*; Appended to an endoglucanase 5A; CAZY family 6; Recombinant (His-tag)	Linear β1,3 and β1,4 glucans; Mixed-linked β1,3-1,4 glucans; Linear β1,2-Glc oligosaccharides with DP-2 and longer	(2, 27)
*Ct*CBM11	*Clostridium thermocellum*; Appended to an endoglucanase; CAZY family 11; Recombinant (His-tag)	Mixed-linked β1,3-1,4 glucans; preference for G4G4G3 repeats	(2, 28, 29)
*Tm*CBM41	*Thermotogoa maritime*; Appended to a pullulanase; CAZY family 41; Recombinant (His-tag)	α1,4 glucans; linear α1,4- and α1,6-linked oligosaccharides	(2, 30)
**Lectins**			
AAL (Aleuria aurantia lectin)	*Aleuria aurantia* mushrooms; Biotinylated; Vector Labs (B-1395)	α-Fuc-linked oligosaccharides (α1,2-; α1,3-; α1,4-; α1,6-)	(31)
ConA (Concanavalin A)	*Canavalia ensiformis* (Jack Bean) seeds; Biotinylated; Vector Labs (B-1005)	α-Man-linked oligosaccharides; Preference for α1,2- and α1,3-Man	(32)
*m*Dectin-1 (Dendritic cell-associated C-type like lectin-1)	Mouse CLEC7A; Recombinant (His-tag); Sino Biological (50233-M07H)	β1,3-Glc oligosaccharide sequences in β1,3-linked glucans	(2, 33)
*h*Malectin	*Homo sapiens*; Recombinant (His-tag)	α-Glc-linked oligosaccharides (α1,3-; α1,4-; α1,6-); diglucosylated high-mannose N-glycans	(34, 35)

Supplemental Table S4. Oligosaccharide probes included in the gluco-oligosaccharide microarray sorted by glycosidic linkage and degree of polymerization (DP), their sequences and the fluorescence binding intensities elicited with BoSGBP$_{MLG}$-A, *Ct*CBM11-His and *Cm*CBM6-2-His.

			**BoSGBP$_{MLG}$-A**	***Ct*CBM11**	***Cm*CBM6-2**
**ID[a]**	**Probe[b]**	**Sequence[c]**	Score±Err[d]	Score±Err[d]	Score±Err[d]
Gluco-delta-fructosides (DP 2-9) – Glc α2					
1	Cyano-2-AO	Glcα-2Glc-AO	-	-	-
2	Cyano-3-AO	Glcα-2Glcα-2Glc-AO	-	-	-
3	Cyano-4-AO	Glcα-2Glcα-2Glcα-2Glc-AO	-	-	-
4	Cyano-5-AO	Glcα-2Glcα-2Glcα-2Glcα-2Glc-AO	-	-	-
5	Cyano-6-AO*	Glcα-2Glcα-2Glcα-2Glcα-2Glcα-2Glc-AO	-	-	-
6	Cyano-7-AO*	Glcα-2Glcα-2Glcα-2Glcα-2Glcα-2Glcα-2Glc-AO	-	-	-
7	Cyano-8-AO*	Glcα-2Glcα-2Glcα-2Glcα-2Glcα-2Glcα-2Glcα-2Glc-AO	-	-	-
8	Cyano-9-AO*	Glcα-2Glcα-2Glcα-2Glcα-2Glcα-2Glcα-2Glcα-2Glcα-2Glc-AO	-	-	-
Poriaco (DP 2-13) – Glc α3					
9	Nigerose-AO	Glcα-3Glc-AO	-	-	-
10	Poria-3-AO	Glcα-3Glcα-3Glc-AO	-	-	-
11	Poria-4-AO	Glcα1-3Glcα1-3Glcα1-3Glc-AO	-	-	-
12	Poria-5-AO	Glcα-3Glcα-3Glcα-3Glcα-3Glc-AO	-	-	-
13	Poria-6-AO	Glcα-3Glcα-3Glcα-3Glcα-3Glcα-3Glc-AO	-	-	-
14	Poria-7-AO	Glcα-3Glcα-3Glcα-3Glcα-3Glcα-3Glcα-3Glc-AO	-	-	-
15	Poria-8-AO*	Glcα-3Glcα-3Glcα-3Glcα-3Glcα-3Glcα-3Glcα-3Glc-AO	-	-	-
16	Poria-9-AO*	Glcα-3Glcα-3Glcα-3Glcα-3Glcα-3Glcα-3Glcα-3Glcα-3Glc-AO	-	-	-
17	Poria-10-AO*	Glcα-3Glcα-3Glcα-3Glcα-3Glcα-3Glcα-3Glcα-3Glcα-3Glα-3Glc-AO	-	-	1136 ±242
18	Poria-11-AO*	Glcα-3Glcα-3Glcα-3Glcα-3Glcα-3Glcα-3Glcα-3Glcα-3Glα-3Glcα-3Glc-AO	-	-	-
19	Poria-12-AO*	Glcα-3Glcα-3Glcα-3Glcα-3Glcα-3Glcα-3Glcα-3Glcα-3Glα-3Glcα-3Glcα-3Glc-AO	-	-	1857 ±33
20	Poria-13-AO*	Glcα-3Glcα-3Glcα-3Glcα-3Glcα-3Glcα-3Glcα-3Glcα-3Glα-3Glcα-3Glcα-3Glcα-3Glc-AO	-	-	-
Maltodextrins (DP 2-13) – Glc α4					
21	Malto-2-AO	Glcα-4Glc-AO	-	-	-
22	Malto-3-AO	Glcα-4Glcα-4Glc-AO	-	-	-
23	Malto-4-AO	Glcα-4Glcα-4Glcα-4Glc-AO	-	-	-
24	Malto-5-AO	Glcα-4Glcα-4Glcα-4Glcα-4Glc-AO	-	-	-
25	Malto-6-AO	Glcα-4Glcα-4Glcα-4Glcα-4Glcα-4Glc-AO	-	-	-
26	Malto-7-AO	Glcα-4Glcα-4Glcα-4Glcα-4Glcα-4Glcα-4Glc-AO	-	-	-
27	Malto-8-AO*	Glcα-4Glcα-4Glcα-4Glcα-4Glcα-4Glcα-4Glcα-4Glc-AO	-	-	-
28	Malto-9-AO*	Glcα-4Glcα-4Glcα-4Glcα-4Glcα-4Glcα-4Glcα-4Glcα-4Glc-AO	-	-	-
29	Malto-10-AO*	Glcα-4Glcα-4Glcα-4Glcα-4Glcα-4Glcα-4Glcα-4Glcα-4Glcα-4Glc-AO	-	-	-
30	Malto-11-AO*	Glcα-4Glcα-4Glcα-4Glcα-4Glcα-4Glcα-4Glcα-4Glcα-4Glcα-4Glcα-4Glc-AO	-	-	-
31	Malto-12-AO*	Glcα-4Glcα-4Glcα-4Glcα-4Glcα-4Glcα-4Glcα-4Glcα-4Glcα-4Glcα-4Glcα-4Glc-AO	-	-	-
32	Malto-13-AO*	Glcα-4Glcα-4Glcα-4Glcα-4Glcα-4Glcα-4Glcα-4Glcα-4Glcα-4Glcα-4Glcα-4Glcα-4Glc-AO	-	-	-

Dextran (DP 2-13) – Glc α6					
33	Dext-2-AO	Glcα-6Glc-AO	-	-	1286 ±287
34	Dext-3-AO	Glcα-6Glcα-6Glc-AO	-	-	-
35	Dext-4-AO	Glcα-6Glcα-6Glcα-6Glc-AO	-	-	-
36	Dext-5-AO*	Glcα-6Glcα-6Glcα-6Glcα-6Glc-AO	-	-	-
37	Dext-6-AO*	Glcα-6Glcα-6Glcα-6Glcα-6Glcα-6Glc-AO	-	-	-
38	Dext-7-AO	Glcα-6Glcα-6Glcα-6Glcα-6Glcα-6Glcα-6Glc-AO	-	-	-
39	Dext-8-AO*	Glcα-6Glcα-6Glcα-6Glcα-6Glcα-6Glcα-6Glcα-6Glc-AO	-	-	-
40	Dext-9-AO*	Glcα-6Glcα-6Glcα-6Glcα-6Glcα-6Glcα-6Glcα-6Glcα-6Glc-AO	-	-	-
41	Dext-10-AO*	Glcα-6Glcα-6Glcα-6Glcα-6Glcα-6Glcα-6Glcα-6Glcα-6Glcα-6Glc-AO	-	-	-
42	Dext-11-AO*	Glcα-6Glcα-6Glcα-6Glcα-6Glcα-6Glcα-6Glcα-6Glcα-6Glcα-6Glcα-6Glc-AO	-	-	-
43	Dext-12-AO*	Glcα-6Glcα-6Glcα-6Glcα-6Glcα-6Glcα-6Glcα-6Glcα-6Glcα-6Glcα-6Glcα-6Glc-AO	-	-	-
44	Dext-13-AO*	Glcα-6Glcα-6Glcα-6Glcα-6Glcα-6Glcα-6Glcα-6Glcα-6Glcα-6Glcα-6Glcα-6Glcα-6Glc-AO	-	-	-
Pullulan (DP 3,4,7) – Glc α4,6					
45	Pano-3-AO	Glcα-6Glcα-4Glc-AO	-	-	-
46	i-Pano-3-AO	Glcα-4Glcα-6Glc-AO	-	-	-
47	Pullu-4-AO	Glcα-6Glcα-4Glcα-4Glc-AO	-	-	-
48	Pullu-6-AO	Glcα-4Glcα-4Glcα-6Glcα-4Glcα-4Glc-AO	-	-	-
49	Pullu-7-AO	Glcα-6Glcα-4Glcα-4Glcα-6Glcα-4Glcα-4Glc-AO	-	-	-
Cyclic β-glucan (DP 2-13) – Glc β2					
50	CβG-2-AO	Glcβ-2Glc-AO	-	-	**25 189 ±1593**
51	CβG-3-AO	Glcβ-2Glcβ-2Glc-AO	-	-	**57 122 ±515**
52	CβG-4-AO	Glcβ-2Glcβ-2Glcβ-2Glc-AO	-	-	**54 499 ±702**
53	CβG-5-AO*	Glcβ-2Glcβ-2Glcβ-2Glcβ-2Glc-AO	-	-	**55 578 ±5248**
54	CβG-6-AO*	Glcβ-2Glcβ-2Glcβ-2Glcβ-2Glcβ-2Glc-AO	-	-	**61 100 ±506**
55	CβG-7-AO*	Glcβ-2Glcβ-2Glcβ-2Glcβ-2Glcβ-2Glcβ-2Glc-AO	-	-	**41 951 ±439**
56	CβG-8-AO*	Glcβ-2Glcβ-2Glcβ-2Glcβ-2Glcβ-2Glcβ-2Glcβ-2Glc-AO	-	-	**51 338 ±1153**
57	CβG-9-AO*	Glcβ-2Glcβ-2Glcβ-2Glcβ-2Glcβ-2Glcβ-2Glcβ-2Glcβ-2Glc-AO	-	-	**49 548 ±202**
58	CβG-10-AO*	Glcβ-2Glcβ-2Glcβ-2Glcβ-2Glcβ-2Glcβ-2Glcβ-2Glcβ-2Glcβ-2Glc-AO	-	-	**49 157 ±1717**
59	CβG-11-AO*	Glcβ-2Glcβ-2Glcβ-2Glcβ-2Glcβ-2Glcβ-2Glcβ-2Glcβ-2Glcβ-2Glcβ-2Glc-AO	-	-	**48 537 ±753**
60	CβG-12-AO*	Glcβ-2Glcβ-2Glcβ-2Glcβ-2Glcβ-2Glcβ-2Glcβ-2Glcβ-2Glcβ-2Glcβ-2Glcβ-2Glc-AO	-	-	**52 583 ±704**
61	CβG-13-AO*	Glcβ-2Glcβ-2Glcβ-2Glcβ-2Glcβ-2Glcβ-2Glcβ-2Glcβ-2Glcβ-2Glcβ-2Glcβ-2Glcβ-2Glc-AO	-	-	**60 491 ±475**
Curdlan (DP 2-13) – Glc β3					
62	Lam-2-AO	Glcβ-3Glc-AO	-	-	**36 030 ±2917**
63	Lam-3-AO	Glcβ-3Glcβ-3Glc-AO	-	-	**5 115 ±197**
64	Lam-4-AO	Glcβ-3Glcβ-3Glcβ-3Glc-AO	-	-	**56 075 ±398**
65	Lam-5-AO	Glcβ-3Glcβ-3Glcβ-3Glcβ-3Glc-AO	-	-	**47 623 ±261**
66	Lam-6-AO*	Glcβ-3Glcβ-3Glcβ-3Glcβ-3Glcβ-3Glc-AO	-	-	**36 235 ±2860**
67	Lam-7-AO	Glcβ-3Glcβ-3Glcβ-3Glcβ-3Glcβ-3Glcβ-3Glc-AO	-	-	**25 160 ±3118**
68	Curd-8-AO*	Glcβ-3Glcβ-3Glcβ-3Glcβ-3Glcβ-3Glcβ-3Glcβ-3Glc-AO	-	-	**48 629 ±551**
69	Curd-9-AO*	Glcβ-3Glcβ-3Glcβ-3Glcβ-3Glcβ-3Glcβ-3Glcβ-3Glcβ-3Glc-AO	-	-	**50 191 ±247**
70	Curd-10-AO*	Glcβ-3Glcβ-3Glcβ-3Glcβ-3Glcβ-3Glcβ-3Glcβ-3Glcβ-3Glcβ-3Glc-AO	-	-	**51 705 ±1610**
71	Curd-11-AO*	Glcβ-3Glcβ-3Glcβ-3Glcβ-3Glcβ-3Glcβ-3Glcβ-3Glcβ-3Glcβ-3Glcβ-3Glc-AO	-	-	**37 489 ±150**
72	Curd-12-AO*	Glcβ-3Glcβ-3Glcβ-3Glcβ-3Glcβ-3Glcβ-3Glcβ-3Glcβ-3Glcβ-3Glcβ-3Glcβ-3Glc-AO	-	-	**48 563 ±712**
73	Curd-13-AO*	Glcβ-3Glcβ-3Glcβ-3Glcβ-3Glcβ-3Glcβ-3Glcβ-3Glcβ-3Glcβ-3Glcβ-3Glcβ-3Glcβ-3Glc-AO	-	-	**33 277 ±56**

Synthetic (DP 8-10) – Glc β3					
74	NSG-11-AO*	Glcβ-3Glcβ-3Glcβ-3Glcβ-3Glcβ-3Glcβ-3Glcβ-3Glcβ-3Glcβ-3Glcβ-3Glc-AO	-	-	**47 959 ± 1058**
75	HE-8-AO	Glcβ-3Glcβ-3Glcβ-3Glcβ-3Glcβ-3Glcβ-3Glcβ-3Glc-AO	-	-	**39 627 ±2500**
76	HE-9-AO	Glcβ-3Glcβ-3Glcβ-3Glcβ-3Glcβ-3Glcβ-3Glcβ-3Glcβ-3Glc-AO	-	-	**20 281 ±644**
77	HE-10-AO	Glcβ-3Glcβ-3Glcβ-3Glcβ-3Glcβ-3Glcβ-3Glcβ-3Glcβ-3Glcβ-3Glc-AO	-	-	**10 143 ±1637**
Cellulose (DP 2-13) – Glc β4					
78	Cellobiose-AO	Glcβ-4Glc-AO	-	-	**19 327 ±2647**
79	Cello-4-AO	Glcβ-4Glcβ-4Glcβ-4Glc-AO	-	-	**23 593 ±2921**
80	Cello-5-AO*	Glcβ-4Glcβ-4Glcβ-4Glcβ-4Glc-AO	-	-	**28 079 ±466**
81	Cello-6-AO*	Glcβ-4Glcβ-4Glcβ-4Glcβ-4Glcβ-4Glc-AO	-	-	**23 282 ±1957**
82	Cello-7-AO*	Glcβ-4Glcβ-4Glcβ-4Glcβ-4Glcβ-4Glcβ-4Glc-AO	-	-	**22 788 ±1546**
83	Cello-8-AO*	Glcβ-4Glcβ-4Glcβ-4Glcβ-4Glcβ-4Glcβ-4Glcβ-4Glc-AO	-	-	**33 768 ±1774**
84	Cello-9-AO*	Glcβ-4Glcβ-4Glcβ-4Glcβ-4Glcβ-4Glcβ-4Glcβ-4Glcβ-4Glc-AO	-	**2 306 ±548**	**49 046 ±831**
85	Cello-10-AO*	Glcβ-4Glcβ-4Glcβ-4Glcβ-4Glcβ-4Glcβ-4Glcβ-4Glcβ-4Glcβ-4Glc-AO	-	**3 250 ±97**	**54 761 ±498**
86	Cello-11-AO*	Glcβ-4Glcβ-4Glcβ-4Glcβ-4Glcβ-4Glcβ-4Glcβ-4Glcβ-4Glcβ-4Glcβ-4Glc-AO	-	**4 396 ±307**	**18 192 ±1294**
87	Cello-12-AO*	Glcβ-4Glcβ-4Glcβ-4Glcβ-4Glcβ-4Glcβ-4Glcβ-4Glcβ-4Glcβ-4Glcβ-4Glcβ-4Glc-AO	-	**2 035 ±558**	**24 965 ±605**
88	Cello-13-AO*	Glcβ-4Glcβ-4Glcβ-4Glcβ-4Glcβ-4Glcβ-4Glcβ-4Glcβ-4Glcβ-4Glcβ-4Glcβ-4Glcβ-4Glc-AO	-	**3 851 ±49**	**57 234 ±1199**
Pustulan (DP 2-15) – Glc β6					
89	Gentiobiose-AO	Glcβ-6Glc-AO	-	-	**18 944 ±359**
90	Pust-3-AO	Glcβ-6Glcβ-6Glc-AO	-	-	**51 332 ±1121**
91	Pust-4-AO	Glcβ-6Glcβ-6Glcβ-6Glc-AO	-	-	**50 271 ±43**
92	Pust-5-AO	Glcβ-6Glcβ-6Glcβ-6Glcβ-6Glc-AO	-	-	**62 706 ±47**
93	Pust-6-AO	Glcβ-6Glcβ-6Glcβ-6Glcβ-6Glcβ-6Glc-AO	-	-	**41 640 ±599**
94	Pust-7-AO*	Glcβ-6Glcβ-6Glcβ-6Glcβ-6Glcβ-6Glcβ-6Glc-AO	-	-	**32 328 ±1258**
95	Pust-8-AO*	Glcβ-6Glcβ-6Glcβ-6Glcβ-6Glcβ-6Glcβ-6Glcβ-6Glc-AO	-	-	**13 006 ±70**
96	Pust-9-AO*	Glcβ-6Glcβ-6Glcβ-6Glcβ-6Glcβ-6Glcβ-6Glcβ-6Glcβ-6Glc-AO	-	-	**24 853 ±1137**
97	Pust-10-AO*	Glcβ-6Glcβ-6Glcβ-6Glcβ-6Glcβ-6Glcβ-6Glcβ-6Glcβ-6Glcβ-6Glc-AO	-	-	**12 164 ±433**
98	Pust-11-AO*	Glcβ-6Glcβ-6Glcβ-6Glcβ-6Glcβ-6Glcβ-6Glcβ-6Glcβ-6Glcβ-6Glcβ-6Glc-AO	-	-	**8 643 ±292**
99	Pust-15-AO*	Glcβ-6Glcβ-6Glcβ-6Glcβ-6Glcβ-6Glcβ-6Glcβ-6Glcβ-6Glcβ-6Glcβ-6Glcβ-6Glcβ-6Glcβ-6Glcβ-6Glc-AO	-	-	**4 772 ±197**
100	Pust-15a-AO*	Glcβ-6Glcβ-6Glcβ-6Glcβ-6Glcβ-6Glcβ-6Glcβ-6Glcβ-6Glcβ-6Glcβ-6Glcβ-6Glcβ-6Glcβ-6Glcβ-6Glc-AO	-	-	**5 825 ±33**
Barley (DP 3-16) – Glc β3,4					
101	Barley-3-AO	Glcβ-4Glcβ-3Glc-AO	-	-	**46 914 ±667**
102	Barley-3a-AO	Glcβ-3Glcβ-4Glc-AO	-	-	**20 036 ±839**
103	Barley-4-AO	Glcβ-4Glcβ-4Glcβ-3Glc-AO	-	-	**48 154 ±6811**
104	Barley-4a-AO	Glcβ-3Glcβ-4Glcβ-4Glc-AO	-	-	**6 152 ±528**
105	Barley-4b-AO	Glcβ-4Glcβ-4Glcβ-3Glc-AO	-	-	**55 745 ±31**
106	Barley-4c-AO	Glcβ-4Glcβ-3Glcβ-4Glc-AO	-	-	**51 168 ±1366**
107	Barley-5-AO*	Glcβ-4Glcβ-4Glcβ-4Glcβ-3Glc-AO	-	**1 298 ±125**	**17 704 ±396**
108	Barley-5a-AO	Glcβ-3Glcβ-4Glcβ-6Glcβ-4Glc-AO	-	-	**53 910 ±2743**
109	Barley-6-AO*	Glcβ-4Glcβ-3Glcβ-4Glcβ-4Glcβ-3Glc-AO	-	**2 339 ±1673**	**41 004 ±767**
110	Barley-6a-AO	Glcβ-3Glcβ-4Glcβ-6Glcβ-4Glcβ-3Glc-AO; and Glcβ-3Glcβ-4Glcβ-4Glcβ-4Glcβ-3Glc-AO (ratio ~1:1)	-	-	**62 618 ±93**
111	Barley-7-AO*	Glcβ-4Glcβ-4Glcβ-3Glcβ-4Glcβ-4Glcβ-3Glc-AO	-	**16 177 ±397**	**52 604 ±693**

112	Barley-8-AO*	(Glcβ-4/3Glcβ)3-4Glcβ-3Glc-AO	-	12 698 ±140	6 754 ±167
113	Barley-9-AO*	(Glcβ-4/3Glcβ)4-3Glc-AO	-	31 593 ±1934	47 212 ±356
114	Barley-10-AO*	(Glcβ-4/3Glcβ)4-4Glcβ-3Glc-AO	-	19 418 ±1431	31 121 ±27
115	Barley-11-AO*	(Glcβ-4/3Glcβ)5-3Glc-AO	-	21 911 ±393	9 037 ±299
116	Barley-12-AO*	(Glcβ-4/3Glcβ)5-4Glcβ-3Glc-AO	-	30 594 ±1510	43 403 ±2187
117	Barley-13-AO*	(Glcβ-4/3Glcβ)6-3Glc-AO	-	18 931 ±4995	28 378 ±673
118	Barley-14-AO*	(Glcβ-4/3Glcβ)6-4Glcβ-3Glc-AO	-	7 868 ±488	34 526 ±1916
119	Barley-15-AO*	(Glcβ-4/3Glcβ)7-3Glc-AO	3 995 ±234	14 544 ±4690	18 333 ±1292
120	Barley-16-AO*	(Glcβ-4/3Glcβ)7-4Glcβ-3Glc-AO	18 621 ±854	28 558 ±436	28 126 ±1853
Grifolan (DP 3-16) – Glc β3/6					
121	Grifo-3-AO*	Glc3(β-3/β-6)-AO	-	-	13 672 ±1649
122	Grifo-4-AO*	Glc4(β-3/β-6)-AO	-	-	54 890 ±1251
123	Grifo-5-AO*	Glc5(β-3/β-6)-AO	-	-	56 938 ±246
124	Grifo-6-AO*	Glc6(β-3/β-6)-AO	-	-	48 062 ±186
125	Grifo-7-AO*	Glc7(β-3/β-6)-AO	-	-	52 521 ±415
126	Grifo-8-AO*	Glc8(β-3/β-6)-AO	-	-	43 565 ±3399
127	Grifo-9-AO*	Glc9(β-3/β-6)-AO	-	-	30 847 ±1208
128	Grifo-10-AO*	Glc10(β-3/β-6)-AO	-	-	31 437 ±2764
129	Grifo-11-AO*	Glc11(β-3/β-6)-AO	-	-	30 643 ±1536
130	Grifo-12-AO*	Glc12(β-3/β-6)-AO	-	-	32 060 ±3979
131	Grifo-13-AO*	Glc13(β-3/β-6)-AO	-	-	31 173 ±3334
132	Grifo-14-AO*	Glc14(β-3/β-6)-AO	-	-	24 016 ±1915
133	Grifo-15-AO*	Glc15(β-3/β-6)-AO	-	-	25 100 ±466
134	Grifo-16-AO*	Glc16(β-3/β-6)-AO	-	-	29 170 ±2011
Lentinan (DP 2-13) – Glc β3/6					
135	Lentin-2-AO	Glc2(β-3/β-6)-AO	-	-	39 179 ±952
136	Lentin-3-AO	Glc3(β-3/β-6)-AO	-	-	23 593 ±144
137	Lentin-4-AO*	Glc4(β-3/β-6)-AO	-	-	46 036 ±659
138	Lentin-5-AO*	Glc5(β-3/β-6)-AO	-	-	62 693 ±35
139	Lentin-6-AO*	Glc6(β-3/β-6)-AO	-	-	10 920 ±243
140	Lentin-7-AO*	Glc7(β-3/β-6)-AO	-	-	59 957 ±2712
141	Lentin-8-AO*	Glc8(β-3/β-6)-AO	-	-	62 698 ±153
142	Lentin-9-AO*	Glc9(β-3/β-6)-AO	-	-	45 493 ±2054
143	Lentin-10-AO*	Glc10(β-3/β-6)-AO	-	-	35 042 ±454
144	Lentin-11-AO*	Glc11(β-3/β-6)-AO	-	-	31 648 ±3029
145	Lentin-12-AO*	Glc12(β-3/β-6)-AO	-	-	24 328 ±832
146	Lentin-13-AO*	Glc13(β-3/β-6)-AO	-	-	49 779 ±1150
Synthetic (DP 6,9-11) – Glc β3/6					
147	HE-9B7-AO	Glcβ-3Glcβ-3Glcβ-3Glcβ-3Glcβ-3Glcβ-3Glcβ-3Glc-AO<br>Glcβ-6	-	-	36 538 ±692
148	HE-10B2-AO	Glcβ-3Glcβ-3Glcβ-3Glcβ-3Glcβ-3Glcβ-3Glcβ-3Glcβ-3Glc-AO<br>Glcβ-6	-	-	48 252 ±58

149	HE-10B3-AO	Glcβ-3Glcβ-3Glcβ-3Glcβ-3Glcβ-3Glcβ-3Glcβ-3Glcβ-3Glc-AO<br>| Glcβ-6	-	-	**50 148 ±1159**
150	HE-10B5-AO	Glcβ-3Glcβ-3Glcβ-3Glcβ-3Glcβ-3Glcβ-3Glcβ-3Glcβ-3Glc-AO<br>| Glcβ-6	-	-	**7 975 ±691**
151	HE-10B7-AO	Glcβ-3Glcβ-3Glcβ-3Glcβ-3Glcβ-3Glcβ-3Glcβ-3Glcβ-3Glc-AO<br>| Glcβ-6	-	-	**9 085 ±394**
152	Gu-6B1/3-AO	Glcβ-3Glcα-3Glcβ-3Glc-AO<br>| | Glcβ-6 Glcβ-6	-	-	**59 710 ±807**
153	HE-11B3/6-AO	Glcβ-3Glcβ-3Glcβ-3Glcβ-3Glcβ-3Glcβ-3Glcβ-3Glcβ-3Glc-AO<br>| | Glcβ-6 Glcβ-6	-	-	**45 410 ±1476**

[a] ID probe position in the microarray matching the position in the binding-charts.

[b] Abbreviations for oligosaccharide moieties: Cyano-, from cyanobacterium gluco-fructosides; Poria-, from a1,3-linked glucan polysaccharide isolated from Poria cocos mycelia; Malto-, from maltodextrins; Dext-, from dextran (MW 200 kDa); Pullu-, from Pullulan; CβG-, from cyclic β1,2-linked glucan isolated from Brucella spp; Lam-, laminarioligosaccharides; Curd-, from Curdlan; Cello-, from cellulose; Pust-, from pustulan; Barley-, from barley glucan; Grifo-, from branched glucan polysaccharide grifolan (95 kDa) isolated from the barmy mycelium of Grifola frondosa; Lenti- from branched glucan polysaccharide lentinan from Lentinus edodes; HE and Gu are synthetic oligosaccharides.

[c] The oligosaccharide probes are all lipid-linked, neoglycolipids (NGLs); AO, NGLs prepared from reducing oligosaccharides by oxime ligation with an aminooxy (AO) functionalized DHPE (1,2-dihexadecyl-sn-glycero-3-phosphoethanolamine) (36).

* Asterisks that follow the names of certain probes indicate that predominant components are shown (2).

[d] Fluorescence binding signals are shown as means of duplicate spots at 5 fmol per spot of each glycan probe with respective error (Err).

- Binding signal below background (<1000).

Supplemental Table S5. Summary of protein-ligand contacts for $BoSGBP_{MLG}$-A-barley-9 (G4G3G4G4G3G4G4G3G) structure. Only chain A is detailed. Distance (d) is expressed in angstroms (Å). Contacts are represented by a double arrow (↔). Aromatic ring (Arom ring). Greek letter delta (δ) denotes O or N atoms from the amino acid sidechain.

**Residue**	**CH-π stacking**	d(Å)	**Direct hydrogen bonds**	d(Å)	**Water-mediated hydrogen bonds**	d(Å)
Ser57			OH↔OH (C1) Glc 1	3.3		
Arg95			NH_2↔OH (C1) Glc 1<br>NH_2↔OH (C2) Glc 1	2.9<br>3.1		
Glu60			COO^-↔OH (C1) Glc 1<br>COO^-↔OH (C2) Glc 1<br>COO^-↔OH (C2) Glc 2	3.3<br>2.2<br>2.9	COO^-↔OH_2 ↔OH_2 ↔OH (C3) Glc 1<br>COO^-↔OH_2 ↔OH_2 ↔OH (C2) Glc 2<br>COO^-↔OH_2 ↔OH_2 ↔OH (C4) Glc 2	3.2;3.1;3.3<br>3.2;3.1;2.7<br>2.9;3.2;3.4
Asn281			NH_2↔OH (C3) Glc 2	3.1	$^{\delta}O$↔OH_2 ↔OH (C3) Glc 1<br>$^{\delta}O$↔OH_2 ↔OH (C2) Glc 2<br>NH_2↔OH_2 ↔OH (C6) Glc 3<br>$^{\delta}O$↔OH_2 ↔OH_2 ↔OH (C6) Glc 3	3.3;3.3<br>3.3;2.7<br>3.5;2.8<br>3.4;2.7;2.8
Tyr266	Arom ring↔Glc 1	4.6			OH↔OH_2 ↔OH_2 ↔OH (C3) Glc 1<br>OH↔OH_2 ↔OH_2 ↔OH (C2) Glc 2	2.9;3.1;3.3<br>2.9;3.1;2.7
Leu56					O↔OH_2 ↔OH_2 ↔OH (C3) Glc 1<br>O↔OH_2 ↔OH_2 ↔OH (C2) Glc 2	3.3;3.1;3.3<br>3.3;3.1;2.7
Arg63			NH_2↔OH (C2) Glc 2	3.5	NH_2↔OH_2 ↔OH_2 ↔OH (C6) Glc 3	3.0;2.7;2.8
Asn78			NH_2↔OH (C3) Glc 2	3.0	NH_2↔OH_2 ↔OH_2 ↔OH (C4) Glc 2<br>$^{\delta}O$↔OH_2 ↔OH (C6) Glc 3	3.0;3.2;3.4<br>2.7;2.8
Tyr82					OH↔OH_2 ↔OH (C4) Glc 2<br>OH↔OH_2 ↔OH_2 ↔OH (C4) Glc 2<br>OH↔OH_2 ↔OH_2 ↔OH (C2) Glc 3	2.7;3.4<br>2.7;2.6;3.3<br>2.7;2.6;2.7
Trp77	Arom ring↔Glc 3<br>Arom ring↔Glc 4	3.6<br>3.8			$^{\delta}NH$↔OH_2 ↔OH_2 ↔OH (C4) Glc 4	2.9;3.4;2.8
Asn64					NH_2↔OH_2 ↔OH_2 ↔OH (C4) Glc 2	3.1;3.2;3.4
Asp282			COO^-↔OH (C6) Glc 3	3.2	O↔OH_2 ↔OH_2 ↔OH (C6) Glc 3<br>COO^-↔OH_2 ↔OH (C2) Glc 4	3.4;2.7;2.8<br>2.6;2.7
Asn286			NH_2↔OH (C2) Glc 4	3.1		
Asn285					$^{\delta}O$↔OH_2 ↔OH (C2) Glc 5<br>O↔OH_2 ↔OH_2 ↔OH (C2) Glc 5<br>O↔OH_2 ↔OH (C2) Glc 5<br>$^{\delta}O$↔OH_2 ↔OH (C3) Glc 5<br>O↔OH_2 ↔OH_2 ↔OH (C3) Glc 5<br>$^{\delta}O$↔OH_2 ↔OH_2 ↔OH (C6) Glc 6	2.9;3.4<br>2.8;3.3;3.4<br>2.8;2.9<br>2.9;2.8<br>2.8;3.3;2.8<br>2.9;2.8;2.7
Arg378					NH_2↔OH_2 ↔OH (C2) Glc 5<br>NH_2↔OH_2 ↔OH_2 ↔OH (C2) Glc 5<br>NH_2↔OH_2 ↔OH (C2) Glc 5<br>NH_2↔OH_2 ↔OH (C3) Glc 5<br>NH_2↔OH_2 ↔OH_2 ↔OH (C3) Glc 5<br>NH_2↔OH_2 ↔OH_2 ↔OH (C6) Glc 6<br>NH_2↔OH_2 ↔OH_2 ↔OH (C6) Glc 6	3.0;3.4<br>2.8;3.3;3.4<br>2.8;2.9<br>3.0;2.8<br>2.8;3.3;2.8<br>3.0;2.9;2.7<br>3.0;2.8;2.7
Trp350	Arom ring↔Glc 5	4.0			$^{\delta}NH$↔OH_2 ↔OH_2 ↔OH (C4) Glc 4<br>$^{\delta}NH$↔OH_2 ↔OH (C6) Glc 5<br>O↔OH_2 ↔OH (C2) Glc 6<br>O↔OH_2 ↔OH_2 ↔OH (C3) Glc 6<br>O↔OH_2 ↔OH_2 ↔OH_2 ↔OH (C6) Glc 7	2.9;2.8;2.8<br>2.9;3.5<br>2.7;2.8<br>2.7;3.1;3.2<br>2.7;3.1;3.4;2.4
Tyr510					OH↔OH_2 ↔OH_2 ↔OH (C6) Glc 5	3.4;2.8;3.5
Glu351					COO^-↔OH_2 ↔OH_2 ↔OH (C6) Glc 5	2.5;2.8;3.5
Arg85					NH_2↔OH_2 ↔OH_2 ↔OH (C6) Glc 5	2.9;2.8;3.5
Trp353	Arom ring↔Glc 6<br>Arom ring↔Glc 7	4.0<br>4.2			$^{\delta}NH$↔OH_2 ↔OH (C6) Glc 8	3.1;3.4
Met376					O↔OH_2 ↔OH_2 ↔OH (C6) Glc 8	2.6;3.2;3.4

Supplemental Table S6. Oligonucleotides used for cloning, re-cloning and site-directed mutagenesis of each construct.

Construct	Oligonucleotide Sequence (5'→3') [a]	Vector
Cloning		
BoSGBP$_{MLG}$-A	(F) TCAGCAAGGGCTGAGGGAATACATGGAAAACATGAATACC<br>(R) TCAGCGGAAGCTGAGGTTAGTTTTCAGTATCCCACCACAG	pHTP1-A57 vector (pET24a derivative); N-terminal 6xHis-tag;
Re-cloning		
BoSGBP$_{MLG}$-A-ZB	(F) TACTTCCAATCCATGATCGACCCGAATGCGCAGCT<br>(R) TATCCACCTTTACTGGTTTTCAGTATCCCACCACAGAAG	pNIC-ZB vector; N-terminal 6xHis-Z-basic tag;
Site-directed mutagenesis		
BoSGBP$_{MLG}$-A*W77A	(F) CAACAACTGATGGGATGT**GCG**AACACCACCAACTACGG<br>(R) CCGTAGTTGGTGGTGTT**CGC**ACATCCCATCAGTTGTTG	Tryptophan 77 mutated to alanine; pHTP1-A57 template;
BoSGBP$_{MLG}$-A*Y266A	(F) CATTTAGTTTCGGACAAGAAGCT**GCC**TCTGATTATCGCGGAAATTCAT<br>(R) ATGAATTTCCGCGATAATCAGA**GGC**AGCTTCTTGTCCGAAACTAAATG	Tyrosine 266 mutated to alanine; pHTP1-A57 template;
BoSGBP$_{MLG}$-A*W350A	(F) CCAGGTGCCTACTCG**GCG**GAACCATGGCCTAC<br>(R) GTAGGCCATGGTTC**CGC**CGAGTAGGCACCTGG	Tryptophan 350 mutated to alanine; pHTP1-A57 template;
BoSGBP$_{MLG}$-A*W353A	(F) GCCTACTCGTGGGAACCA**GCG**CCTACAGGG<br>(R) CCCTGTAGG**CGC**TGGTTCCCACGAGTAGGC	Tryptophan 353 mutated to alanine; pHTP1-A57 template;
BoSGBP$_{MLG}$-A*R378A	(F) CGTTACAGCAACCATGGCT**GCT**GAAGTGGAACCCAAA<br>(R) TTTGGGTTCCACTTC**AGC**AGCCATGGTTGCTGTAACG	Arginine 378 mutated to alanine; pHTP1-A57 template;

[a] Forward primer (F) and Reverse primer (R); LIC vector complementary sequences are underlined; Mutated base pairs are highlighted in bold;

Supplemental Table S7. Supplementary glycan microarray document based on MIRAGE Guidelines (doi:10.3762/mirage.3) (37).

Classification	Description
1. Sample: Glycan Binding Sample	
Description of Sample	Sample name: BoSGBP$_{MLG}$-A, SusD-like protein from *Bacteroides ovatus* ATCC 8483;<br>Origin: Recombinant<br>Method of preparation: As described in the *Materials and Methods* section of the main text.
Sample modifications	Not relevant.
Assay protocol	Please see *Materials and Methods* section in the main text.
2. Glycan Library	
Glycan description for defined glycans	Two carbohydrate microarrays were used:<br>1) The "Fungal, bacterial, microalgae and plant saccharide microarray" comprised 31 saccharides (polysaccharides and glycoproteins) derived from fungi, bacteria, and plants. Some of the polysaccharides have been described previously (2). Some were from commercial sources or were from collaborators prepared in their laboratories: purified *Candida albicans* N-linked mannoprotein preparation from David Williams (East Tennessee State University); xyloglucan and xylan fractions from plum, arabinoxylan from brewers' spent grain and the *Nannochloropsis oculata* mixed-linkage β1,3-1,4 glucan enriched fraction from University of Aveiro (Portugal). The antigen preparations from *Mycobacterium tuberculosis* were obtained from the NIH Biodefense and Emerging Infections Research Resources Repository (Beiresources). The predominant oligosaccharide sequences, sources and references for their preparation, when available, are in Table S1.<br>2) The "Gluco-oligosaccharide microarray" comprised 153 sequence-defined gluco-oligosaccharides prepared as neoglycolipid (NGL) probes. These were described earlier (2). The probe name and sequences are listed in Table S4.
Glycan description for undefined glycans	Not relevant.
Glycan modifications	Polysaccharides and glycoproteins were not modified.<br>The NGLs were prepared from reducing oligosaccharides by oxime ligation with an aminooxy-functionalized 1,2-dihexadecyl-*sn*-glycero-3-phosphoethanolamine (DHPE) [(AOPE) (36).
3. Printing Surface; e.g., Microarray Slide	
Description of surface	Nitrocellulose-coated glass microarray slides.
Manufacturer	16-pad UniSart® 3D Microarray Slide from Sartorius (Goettingen, Germany)
Custom preparation of surface	Not relevant.
Non-covalent Immobilization	Polysaccharides and glycoproteins were immobilized non-covalently without any formulation. For construction of the 'Fungal, bacterial, microalgae and plant saccharide microarray', polysaccharides and glycoproteins were taken up in water, except for curdlan polysaccharide that was solubilised using mild alkaline solution (50mM NaOH) and glucurono-xylomannan solubilised in 150mM NaCl.

	The lipid-linked oligosaccharide probes were formulated as liposomes by adding carrier lipids, 1,2-dihexanoyl-sn-glycero-3-phosphocholine (DHPC) and cholesterol for arraying and non-covalent immobilisation on nitrocellulose-coated glass slides (38).
4. Arrayer (Printer)	
Description of Arrayer	Nano-Plotter 2.1 (GeSiM, Radeberg, Germany)
Dispensing mechanism	Non-contact liquid delivery with four dispensing tips.
Glycan deposition	Approximately 0.33 nL was printed per spot.<br>Polysaccharides and glycoproteins were printed at two levels, 0.03 and 0.1 ng per spot; and NGLs at 2 and 5 fmol per spot; all in duplicate.
Printing conditions	The printing solutions were aqueous-based. Printing was performed at room temperature and relative humidity of 58%.<br>The printing solutions of polysaccharides and glycoproteins were at 0.1 and 0.3 mg/mL for the 0.03 and 0.1 ng per spot levels, respectively.<br>The NGL printing solutions contained 100 pmol/μl of DHPC and cholesterol (both from SIGMA) as lipid carriers in addition to the lipid-linked glycan probes. The concentrations of the NGL probes were 5 and 15 pmol/μl for the 2 and 5 fmol per spot levels, respectively.<br>The printing solutions also contained Cy3 NHS ester (GE Healthcare) at 20 ng/mL (26 fmol/μL) as a marker to monitor the printing process.
5. Glycan Microarray with “Map”	
Array layout	The arrayed slides contained 16 identical pads (subarrays). Each pad was set up for printing 64 probes maximum, each at 2 levels in duplicate (four spots for one probe in a row); 256 spots (16x16) in total for 64 probes. When not completely printed, the remaining space in each pad was treated as “blank” to allow quantification using the same grid; “blank probes” were excluded from final data presentation.
Glycan identification and quality control	The Fungal, bacterial, microalgae and plant saccharide microarray was analysed with 18 sequence-specific proteins for data validation. These included: 1) Monoclonal antibodies – BS400-2 (β1,3-Glucan), BS400-3 (β1,3/1,4-Glucan), LM5 (β1,4-Galactan), BS400-4 (β1,4-Mannan), LM6 (α1,5-Arabinan), LM21 (β1,4-Heteromannan), LM11 (β1,4-Xylan/Arabinoxylan) and LM25 (Xyloglucan); 2) Carbohydrate-binding modules of bacterial glycoside hydrolases with specificity for α- and β-glucans – *Ct*CBM11 and *Tm*CBM41; 3) and Lectins – *Aleuria aurantia* lectin (AAL), Concanavalin A (ConA), murine Dendritic cell-associated C-type like lectin-1 (mDectin-1) and human Malectin to target α-Fuc, α-Man, β1,3-Glc and α-Glc-linked oligosaccharides, respectively.<br>The gluco-oligosaccharide microarray was analysed with the carbohydrate-binding modules *Ct*CBM11 and *Cm*CBM6-2, both with proven specificity for mixed-linkage β1,3/1,4 glucans.<br>All validation proteins and described specificity are listed in Table S3. Predicted binding signals were recorded.
6. Detector and Data Processing	
Scanning hardware	GenePix 4300A (Molecular Devices, UK)
Scanner settings	Scanning resolution: 10 μm / pixel (this resolution is adequate for the sizes of sample spots)<br>Laser channel: Red (scan wavelength 635 nm)<br>PMT: 350<br>Scan powers: 5-90% to achieve maximum unsaturated fluorescence signal

Image analysis software	GenePix® Pro 7 (Molecular Devices, UK)
Data processing	The .gpr file was inserted into an in-house microarray database using software for data processing (designed by Mark Stoll, http://www.beilstein-institut.de/en/publications/proceedings/glyco-2009). No particular normalisation method or statistical analysis was used.
7. Glycan Microarray Data Presentation	
Data presentation	The microarray binding results are in **Figures 2** and **3** and in **Tables S2** and **S4**.
8. Interpretation and Conclusion from Microarray Data	
Data interpretation	Software or algorithms were not used to interpret processed data.
Conclusions	In the Fungal, bacterial, microalgae and plant saccharide microarray, BoSGBP$_{MLG}$-A bound strongly to mixed-linkage β1,3/1,4-glucans of different sources. BoSGBP$_{MLG}$-A also showed binding, albeit with lower intensities, to xyloglucan fractions that have a β1,4-linked glucose backbone.<br><br>In the gluco-oligosaccharide microarray, binding of BoSGBP$_{MLG}$-A was restricted to longer mixed-linkage β1,3/1,4- gluco oligosaccharides derived from barley (DP15 and DP16, probes 119-120). The requirement of a minimum chain length for detecting binding suggested that internal sequence of the oligosaccharide was important for recognition.

Supplemental Table S8. Privateer validation report of $BoSGBP_{MLG}$-A in complex with barley-9 oligosaccharide (39).

Chain	Name	Q[1]	Phi	Theta	Anomer	D/L[2]	Conformation	RSCC[3]	Average B-factor	Diagnostic
A	BGC-1	0.54	307.43	15.40	beta	D	$^4C_1$	0.68	61.56	Ok
A	BGC-2	0.53	88.60	19.41	beta	D	$^4C_1$	0.88	32.63	Ok
A	BGC-3	0.53	211.08	2.48	beta	D	$^4C_1$	0.89	23.51	Ok
A	BGC-4	0.53	352.55	8.14	beta	D	$^4C_1$	0.88	22.90	Ok
A	BGC-5	0.50	163.57	4.75	beta	D	$^4C_1$	0.86	24.79	Ok
A	BGC-6	0.54	74.15	4.06	beta	D	$^4C_1$	0.90	24.63	Ok
A	BGC-7	0.57	356.86	11.62	beta	D	$^4C_1$	0.88	28.65	Ok
A	BGC-8	0.57	357.02	8.71	beta	D	$^4C_1$	0.78	39.37	Ok
A	BGC-9	0.55	103.44	1.88	beta	D	$^4C_1$	0.55	52.57	Ok
B	BGC-1	0.49	310.59	16.57	beta	D	$^4C_1$	0.70	52.47	Ok
B	BGC-2	0.59	33.42	7.93	beta	D	$^4C_1$	0.83	34.25	Ok
B	BGC-3	0.57	115.37	4.58	beta	D	$^4C_1$	0.86	23.16	Ok
B	BGC-4	0.59	327.20	6.08	beta	D	$^4C_1$	0.88	21.66	Ok
B	BGC-5	0.57	75.47	5.51	beta	D	$^4C_1$	0.87	23.58	Ok
B	BGC-6	0.61	353.45	3.04	beta	D	$^4C_1$	0.87	22.55	Ok
B	BGC-7	0.56	2.95	5.94	beta	D	$^4C_1$	0.85	27.59	Ok
B	BGC-8	0.62	4.30	8.10	beta	D	$^4C_1$	0.74	36.59	Ok
B	BGC-9	0.53	143.17	7.61	beta	D	$^4C_1$	0.60	50.36	Ok

[1] Q is the total puckering amplitude, measured in Å. [2] Molecule's stereochemistry or chiral handedness. [3] RSCC, Real Space Correlation Coefficient, measures the agreement between model and positive omit density where RSCC < 0.8 is typically considered poor.

SUPPLEMENTAL REFERENCES

1. Haworth WN, Hirst EL, Isherwood FA. 1937. Polysaccharides. Part XXIV. Yeast mannan. J Chem Soc 160:784–791.
2. Palma AS, Liu Y, Zhang H, Zhang Y, McCleary B V., Yu G, Huang Q, Guidolin LS, Ciocchini AE, Torosantucci A, Wang D, Carvalho AL, Fontes CMGA, Mulloy B, Childs RA, Feizi T, Chai W. 2015. Unravelling Glucan Recognition Systems by Glycome Microarrays Using the Designer Approach and Mass Spectrometry. Mol Cell Proteomics 14:974–988.
3. Mccleary B V., Matheson NK. 1986. Enzymic Analysis of Polysaccharide Structure. Adv Carbohydr Chem Biochem 44:147–276.
4. Zhang H-T, Zhan X-B, Zheng Z-Y, Wu J-R, English N, Yu X-B, Lin C-C. 2012. Improved curdlan fermentation process based on optimization of dissolved oxygen combined with pH control and metabolic characterization of Agrobacterium sp. ATCC 31749. Appl Microbiol Biotechnol 93:367–379.
5. Hong F, Hansen RD, Yan J, Allendorf DJ, Baran JT, Ostroff GR, Ross GD. 2003. β-Glucan Functions as an Adjuvant for Monoclonal Antibody Immunotherapy by Recruiting Tumoricidal Granulocytes as Killer Cells. Cancer Res 63:9023–9031.
6. Jamas S, Easson DD, Ostroff GR, Onderdonk AB. 1991. PGG-Glucans: A Novel Class of Macrophage-Activating Immunomodulators. Polym Drugs Drug Deliv Syst Vol 469 44–51.
7. Wang X, Xu X, Zhang L. 2008. Thermally Induced Conformation Transition of Triple-Helical Lentinan in NaCl Aqueous Solution. J Phys Chem B 112:10343–10351.
8. Du Y, Gu G, Hua Y, Wei G, Ye X, Yu G. 2004. Synthesis and antitumor activities of glucan derivatives. Tetrahedron 60:6345–6351.
9. De la Cruz J, Pintor-Toro JA, Benitez T, Llobell A. 1995. Purification and characterization of an endo-β-1,6-glucanase from Trichoderma harzianum that is related to its mycoparasitism. J Bacteriol 177:1864–1871.
10. Yoo D-H, Lee B-H, Chang P-S, Lee HG, Yoo S-H. 2007. Improved Quantitative Analysis of Oligosaccharides from Lichenase-Hydrolyzed Water-Soluble Barley β-Glucans by High-Performance Anion-Exchange Chromatography. J Agric Food Chem 55:1656–1662.
11. Rudkin FM, Raziunaite I, Workman H, Essono S, Belmonte R, MacCallum DM, Johnson EM, Silva LM, Palma AS, Feizi T, Jensen A, Erwig LP, Gow NAR. 2018. Single human B cell-derived monoclonal anti-Candida antibodies enhance phagocytosis and protect against disseminated candidiasis. Nat Commun 9:5288.
12. Pandeirada CO, Maricato É, Ferreira SS, Correia VG, Pinheiro BA, Evtuguin D V., Palma AS, Correia A, Vilanova M, Coimbra MA, Nunes C. 2019. Structural analysis and potential immunostimulatory activity of Nannochloropsis oculata polysaccharides. Carbohydr Polym 222:114962.
13. Nunes C, Saraiva JA, Coimbra MA. 2008. Effect of candying on cell wall polysaccharides of plums (Prunus domestica L.) and influence of cell wall enzymes. Food Chem 111:538–548.
14. Coelho E, Rocha MAM, Moreira ASP, Domingues MRM, Coimbra MA. 2016. Revisiting the structural features of arabinoxylans from brewers' spent grain. Carbohydr Polym 139:167–176.

15. Hirabayashi J, Tateno H, Takahara K, Inaba K, Shibata N, Arita T, Okawa Y, Tokieda S. 2012. Difference in Fine Specificity to Polysaccharides of Candida albicans Mannoprotein between Mouse SIGNR1 and Human DC-SIGN. Infect Immun 80:1699–1706.

16. Hanashima S, Götze S, Liu Y, Ikeda A, Kojima-Aikawa K, Taniguchi N, Varón Silva D, Feizi T, Seeberger PH, Yamaguchi Y. 2015. Defining the Interaction of Human Soluble Lectin ZG16p and Mycobacterial Phosphatidylinositol Mannosides. ChemBioChem 16:1502–1511.

17. Mishra AK, Driessen NN, Appelmelk BJ, Besra GS. 2011. Lipoarabinomannan and related glycoconjugates: structure, biogenesis and role in Mycobacterium tuberculosis physiology and host–pathogen interaction. FEMS Microbiol Rev 35:1126–1157.

18. Dobos KM, Khoo KH, Swiderek KM, Brennan PJ, Belisle JT. 1996. Definition of the full extent of glycosylation of the 45-kilodalton glycoprotein of Mycobacterium tuberculosis. J Bacteriol 178:2498–2506.

19. Meikle PJ, Bonig I, Hoogenraad NJ, Clarke AE, Stone BA. 1991. The location of (1→3)-β-glucans in the walls of pollen tubes of Nicotiana alata using a (1→3)-β-glucan-specific monoclonal antibody. Planta 185:1–8.

20. Meikle PJ, Hoogenraad NJ, Bonig I, Clarke AE, Stone BA. 1994. A (1→3,1→4)-β-glucan-specific monoclonal antibody and its use in the quantitation and immunocytochemical location of (1→3,1→4)-β-glucans. Plant J 5:1–9.

21. Pettolino FA, Hoogenraad NJ, Ferguson C, Bacic A, Johnson E, Stone BA. 2001. A (1→4)-β-mannan-specific monoclonal antibody and its use in the immunocytochemical location of galactomannans. Planta 214:235–242.

22. Jones L, Seymour GB, Knox JP. 1997. Localization of Pectic Galactan in Tomato Cell Walls Using a Monoclonal Antibody Specific to (1→4)-β-D-Galactan. Plant Physiol 113:1405–1412.

23. Willats WGT, Marcus SE, Knox JP. 1998. Generation of a monoclonal antibody specific to (1→5)-α-l-arabinan. Carbohydr Res 308:149–152.

24. McCartney L, Marcus SE, Knox JP. 2005. Monoclonal Antibodies to Plant Cell Wall Xylans and Arabinoxylans. J Histochem Cytochem 53:543–546.

25. Marcus SE, Blake AW, Benians TAS, Lee KJD, Poyser C, Donaldson L, Leroux O, Rogowski A, Petersen HL, Boraston A, Gilbert HJ, Willats WGT, Paul Knox J. 2010. Restricted access of proteins to mannan polysaccharides in intact plant cell walls. Plant J 64:191–203.

26. Pedersen HL, Fangel JU, McCleary B, Ruzanski C, Rydahl MG, Ralet MC, Farkas V, Von Schantz L, Marcus SE, Andersen MCF, Field R, Ohlin M, Knox JP, Clausen MH, Willats WGT. 2012. Versatile high resolution oligosaccharide microarrays for plant glycobiology and cell wall research. J Biol Chem 287:39429–39438.

27. Henshaw JL, Bolam DN, Pires VMR, Czjzek M, Henrissat B, Ferreira LMA, Fontes CMGA, Gilbert HJ. 2004. The Family 6 Carbohydrate Binding Module Cm CBM6-2 Contains Two Ligand-binding Sites with Distinct Specificities. J Biol Chem 279:21552–21559.

28. Carvalho AL, Goyal A, Prates JAM, Bolam DN, Gilbert HJ, Pires VMR, Ferreira LMA, Planas A, Romão MJ, Fontes CMGA. 2004. The family 11 carbohydrate-binding module of Clostridium thermocellum Lic26A-Cel5E accommodates β-1,4- and β-1,3-1,4-mixed linked glucans at a single binding site. J Biol Chem 279:34785–34793.

29. Ribeiro D, Viegas A, Pires V, Medeiros-Silva J, Bule P, Wengang C, Marcelo F, Fontes CMGA, Cabrita E, Palma A, Carvalho AL. 2020. Molecular basis for the preferential recognition of β1,3-1,4-Glucans by the Family 11 Carbohydrate-Binding Module from Clostridium thermocellum. FEBS J 287:2723–2743.

30. Lammerts Van Bueren A, Finn R, Ausió J, Boraston AB. 2004. α-Glucan recognition by a new family of carbohydrate-binding modules found primarily in bacterial pathogens. Biochemistry 43:15633–15642.

31. Bergström M, Åström E, Påhlsson P, Ohlson S. 2012. Elucidating the selectivity of recombinant forms of Aleuria aurantia lectin using weak affinity chromatography. J Chromatogr B Anal Technol Biomed Life Sci 885–886:66–72.

32. Wang L, Cummings RD, Smith DF, Huflejt M, Campbell CT, Gildersleeve JC, Gerlach JQ, Kilcoyne M, Joshi L, Serna S, Reichardt NC, Pera NP, Pieters RJ, Eng W, Mahal LK. 2014. Cross-platform comparison of glycan microarray formats. Glycobiology 24:507–517.

33. Palma AS, Feizi T, Zhang Y, Stoll MS, Lawson AM, Díaz-Rodríguez E, Campanero-Rhodes MA, Costa J, Gordon S, Brown GD, Chai W. 2006. Ligands for the β-glucan receptor, dectin-1, assigned using “designer” microarrays of oligosaccharide probes (neoglycolipids) generated from glucan polysaccharides. J Biol Chem 281:5771–5779.

34. Schallus T, Jaeckh C, Fehér K, Palma AS, Liu Y, Simpson JC, Mackeen M, Stier G, Gibson TJ, Feizi T, Pieler T, Muhle-Goll C. 2008. Malectin: A Novel Carbohydrate-binding Protein of the Endoplasmic Reticulum and a Candidate Player in the Early Steps of Protein N - Glycosylation. Mol Biol Cell 19:3404–3414.

35. Palma AS, Liu Y, Muhle-Goll C, Butters TD, Zhang Y, Childs R, Chai W, Feizi T. 2010. Multifaceted Approaches Including Neoglycolipid Oligosaccharide Microarrays to Ligand Discovery for Malectin, p. 265–286. In Fukuda, M (ed.), Methods in Enzymology, vol. 478, 1st ed. Elsevier Inc, Amsterdam.

36. Liu Y, Feizi T, Campanero-Rhodes MA, Childs RA, Zhang Y, Mulloy B, Evans PG, Osborn HMI, Otto D, Crocker PR, Chai W. 2007. Neoglycolipid Probes Prepared via Oxime Ligation for Microarray Analysis of Oligosaccharide-Protein Interactions. Chem Biol 14:847–859.

37. Liu Y, McBride R, Stoll M, Palma AS, Silva L, Agravat S, Aoki-Kinoshita KF, Campbell MP, Costello CE, Dell A, Haslam SM, Karlsson NG, Khoo K-H, Kolarich D, Novotny M V., Packer NH, Ranzinger R, Rapp E, Rudd PM, Struwe WB, Tiemeyer M, Wells L, York WS, Zaia J, Kettner C, Paulson JC, Feizi T, Smith DF. 2017. The minimum information required for a glycomics experiment (MIRAGE) project: improving the standards for reporting glycan microarray-based data. Glycobiology 27:280–284.

38. Liu Y, Childs RA, Palma AS, Campanero-Rhodes MA, Stoll MS, Chai W, Feizi T. 2012. Neoglycolipid-Based Oligosaccharide Microarray System: Preparation of NGLs and Their Noncovalent Immobilization on Nitrocellulose-Coated Glass Slides for Microarray Analyses, p. 117–136. *In* Chevolot, Y (ed.), Methods in Molecular Biology, vol. 808, 1st ed. Humana Press, Totowa, NJ.

39. Agirre J, Iglesias-Fernández J, Rovira C, Davies GJ, Wilson KS, Cowtan KD. 2015. Privateer: software for the conformational validation of carbohydrate structures. Nat Struct Mol Biol 22:833–834.
